# Supplementary material for: Flexibility of intrinsic neural timescales during distinct behavioral states
Source: Commun Biol. 2024 Dec 19;7:1667. doi: 10.1038/s42003-024-07349-1 (PMC11659614; doi:10.1038/s42003-024-07349-1)
Supplement: Supplementary file 1 — Supplementary Material [file 42003_2024_7349_MOESM1_ESM.pdf]

# Supplementary Material

## Timescale Values Across the Brain in Mice

In the figure below, we show the values of $\tau$ across the brain in all of the mice. The $\tau$ values were averaged across windows of activity (see methods).

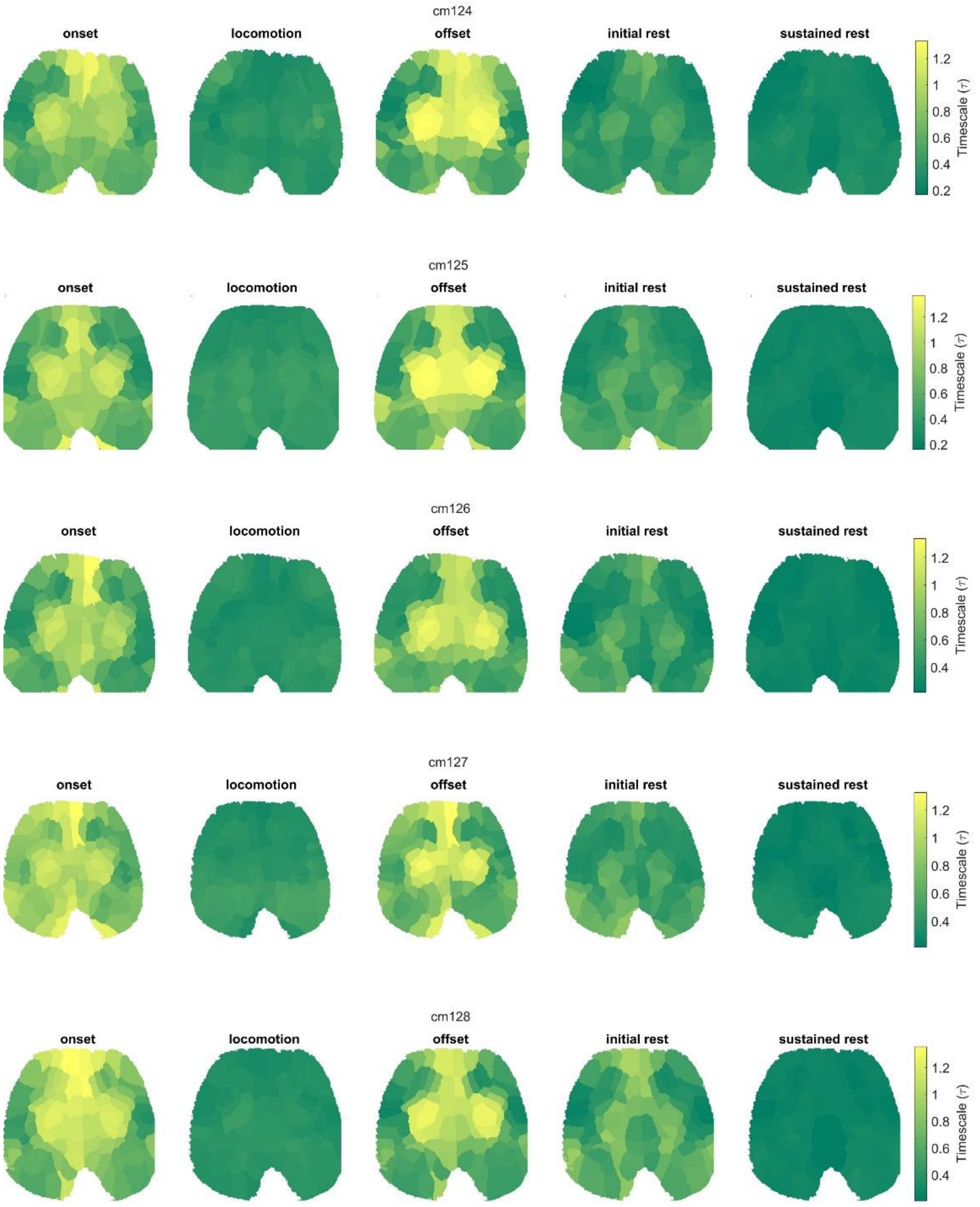


*Supplementary Figure 1: The distribution of averaged ACW-0 values across 92 ROIs for each of the mice.*

## ROC Curves and Confusion Matrices for Each Fold for Mice Data

We used nested cross-validation for hyperparameter optimization of our support vector machines. 10 inner and 10 outer folds were used. The hyperparameter optimization was done on the inner folds using 10-fold cross validation, then the model was tested on the outer fold. Here, we report the confusion matrices and ROC curves for each of the tests on 10 outer folds for the mice data.

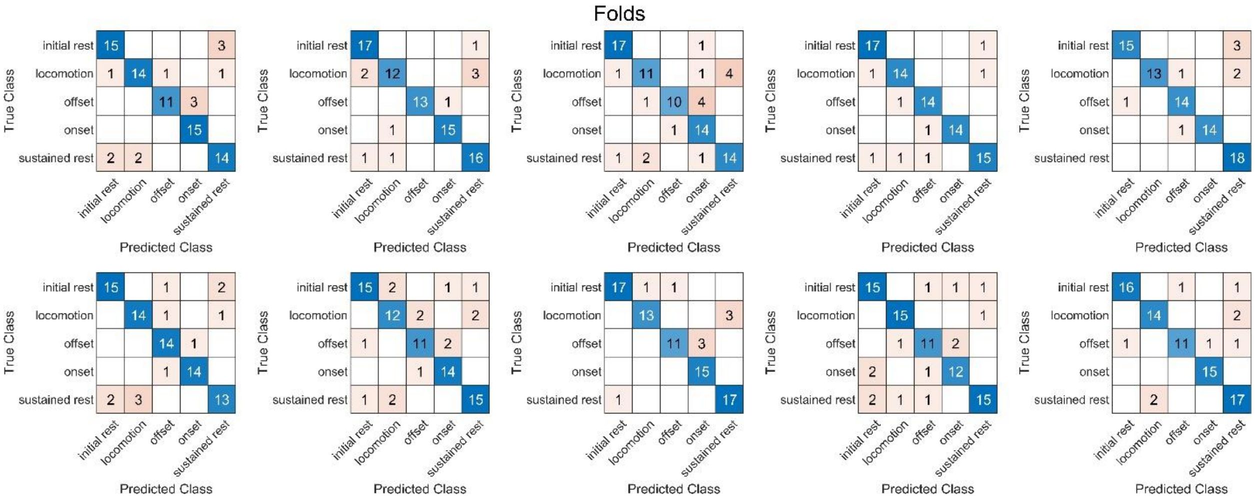


*Supplementary Figure 2. Confusion matrices for each of the 10 outer folds (see above text and methods) for the mice calcium imaging data.*

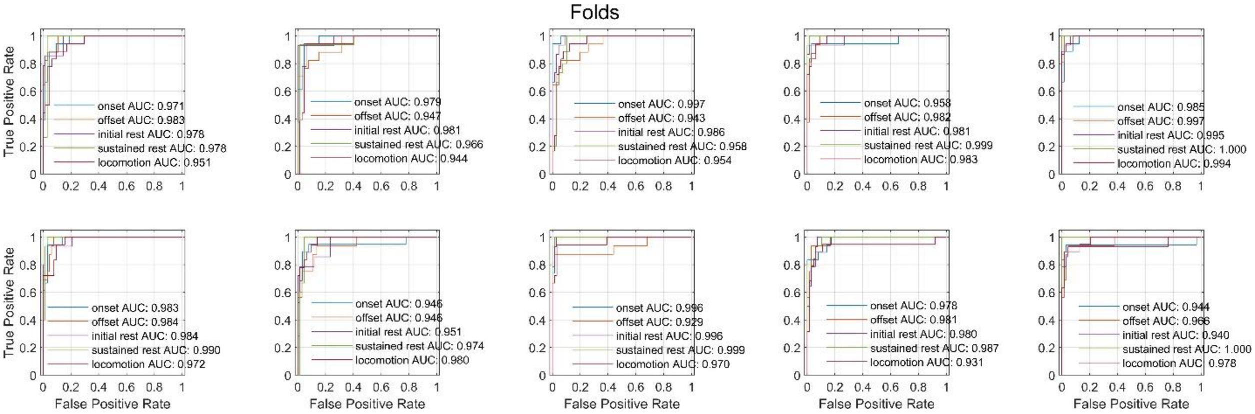


*Supplementary Figure 3. ROC curves for each of the 10 outer folds (see above text and methods) for the mice calcium imaging data. AUC: Area under the curve.*

## *Replication of ROC Curves and Confusion Matrices in Mice Data using ACW – 0*

We replicated the main analyses in the paper using ACW – 0: the lag where autocorrelation function reaches 0 instead of $\tau$. We started our analyses with the machine learning analyses for mice behavioral classification using SVM. Supplementary figure 4A shows ACW – 0 values across the mice brain in one of the mice. The confusion matrix for test data aggregated across folds can be seen in figure supplementary figure 4B. In all behavioral states, SVM performed higher than random chance (20% for 5 states), between 68.7% for locomotion and 81.3% for sustained rest; and showed an overall accuracy of 75.7%. The one-versus-all ROC curve averaged across folds is given in supplementary figure 4C, showing that for every state, SVM reached an AUC of more than 0.9, indicating that false positives and false negatives are relatively low. The results for individual folds can be found in supplementary figures 5 and 6.

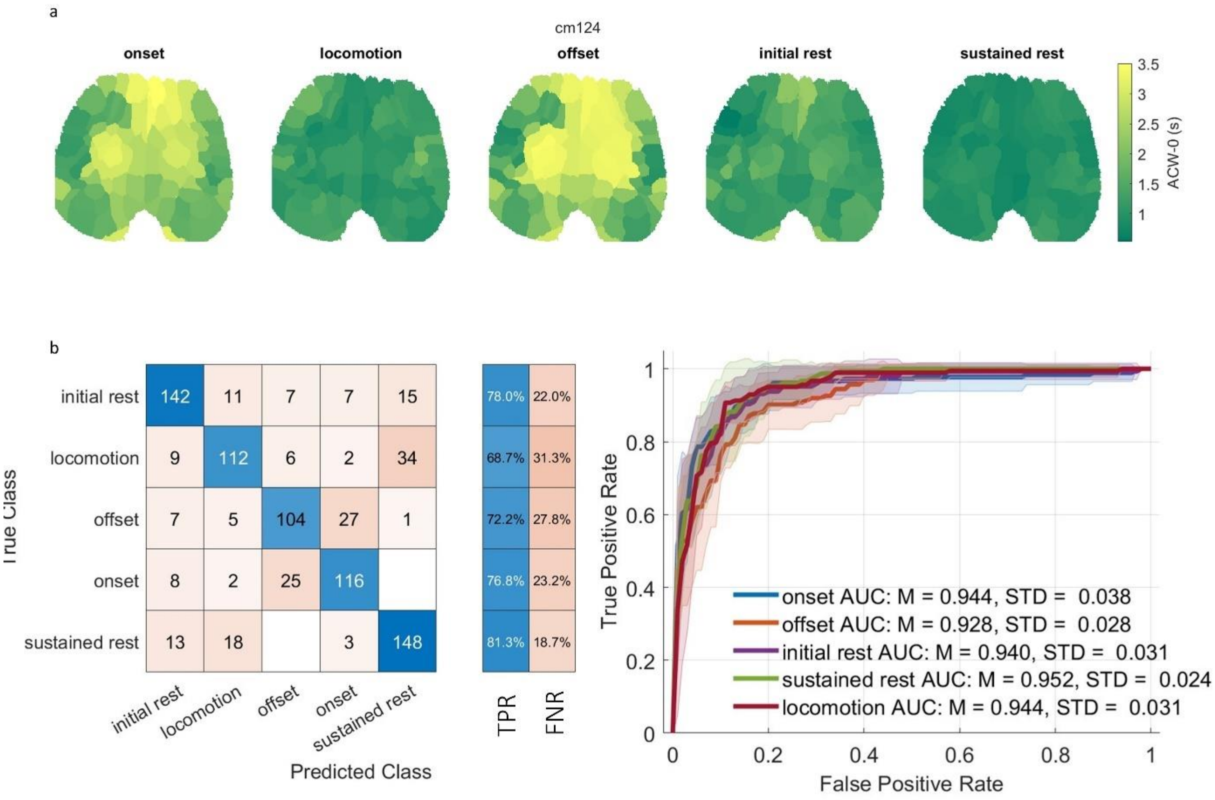


*Supplementary Figure 4. Classification learning for behavioral states using ACW – 0. a. The averaged ACW-0 values across the brain for one of the mice. The same figure for other mice can be found in supplementary figure 1. b. We trained support vector machines to classify the behavioral states of mice based on the ACW-0 values across the brain with nested cross validation for hyperparameter tuning (10 inner, 10 outer folds). Panel B shows the confusion matrix for test data, aggregated across folds. C. Receiver operating characteristic curve for the test data using the trained support vector machine. The data ROC curves from 10 folds were averaged and the standard deviation across the folds were indicated as shading. Abbreviations: TPR: True positive rate, FNR: False negative rate, AUC: Area under the curve, M: Mean, STD: Standard deviation*

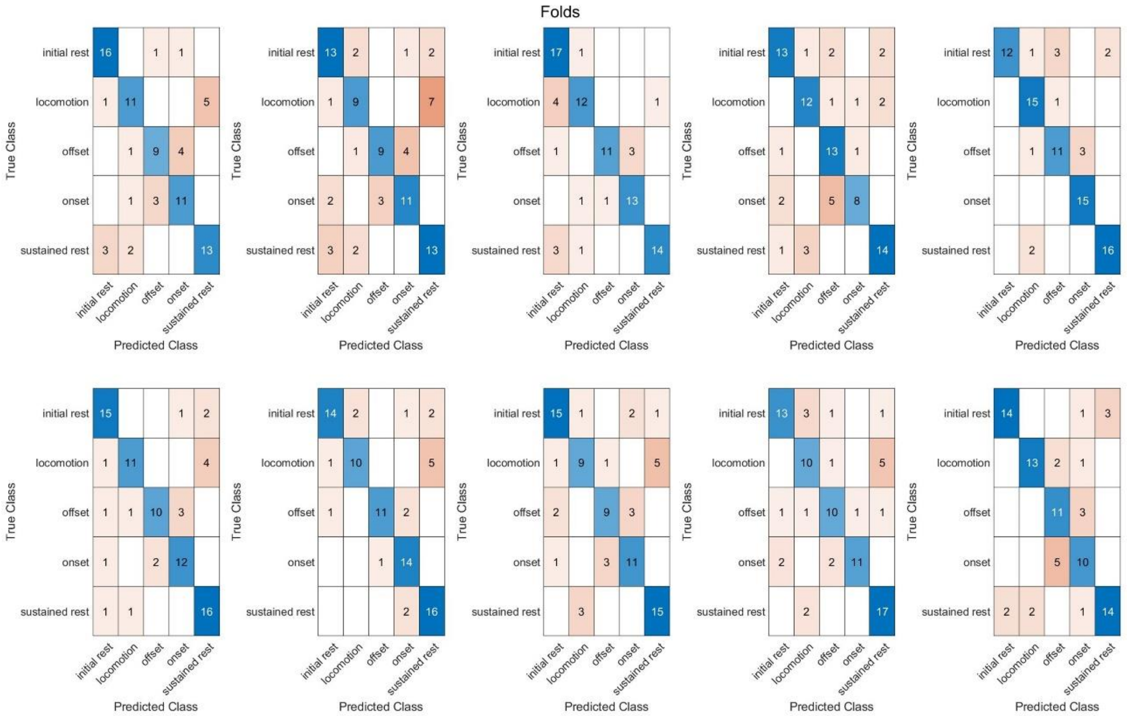


*Supplementary Figure 5. Confusion matrices for each of the 10 outer folds (see above text and methods) for the mice calcium imaging data using ACW – 0 instead of $\tau$.*

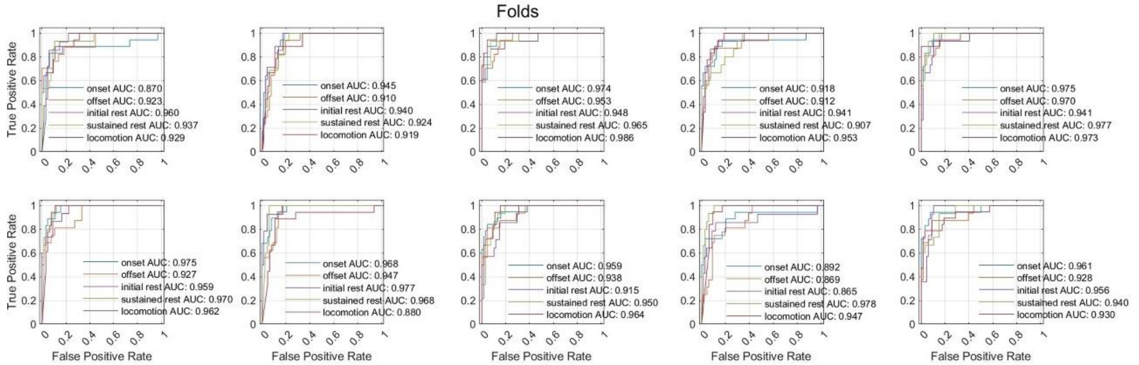


*Supplementary Figure 6. ROC curves for each of the 10 outer folds (see above text and methods) for the mice calcium imaging data using ACW – 0 instead of $\tau$. AUC: Area under the curve.*

## Logistic Regression Results for Mice Data

In addition to SVM, we performed logistic regression for prediction of behavioral states from data. Supplementary figure 7 shows the average ROC curve and aggregate confusion matrix evaluated on the test set. Supplementary figures 8 and 9 show individual ROC curves and confusion matrices for each fold.

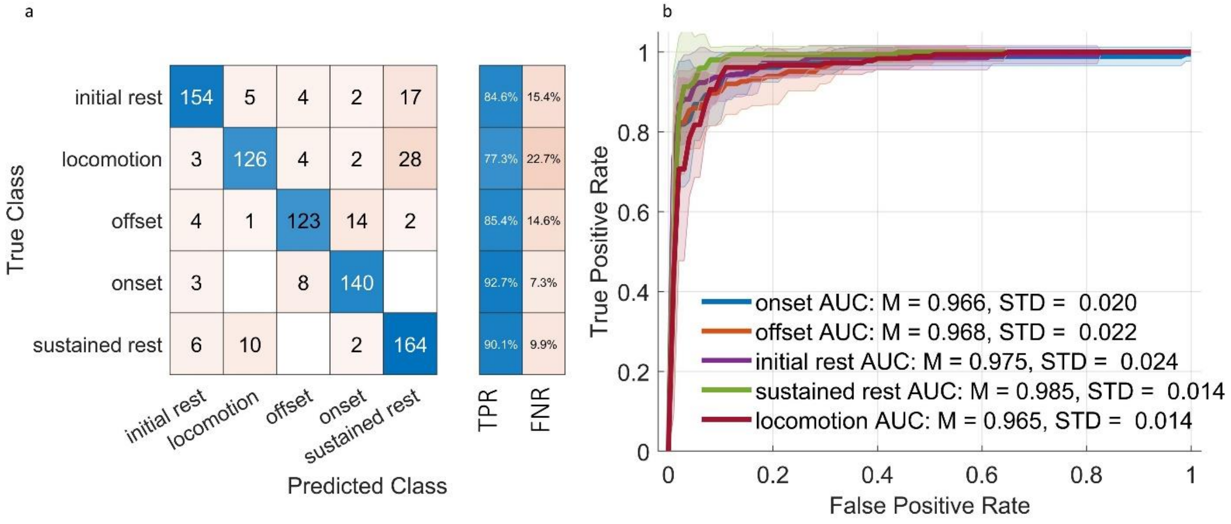


*Supplementary Figure 7. Classification learning for behavioral states using logistic regression instead of SVM. a. We trained logistic regression models to classify the behavioral states of mice based on the ACW-0 values across the brain with nested cross validation for hyperparameter tuning (10 inner, 10 outer folds). Panel a shows the confusion matrix for test data, aggregated across folds. b. Receiver operating characteristic curve for the test data using the logistic regression model. The data ROC curves from 10 folds were averaged and the standard deviation across the folds were indicated as shading. Abbreviations: TPR: True positive rate, FNR: False negative rate, AUC: Area under the curve, M: Mean, STD: Standard deviation*

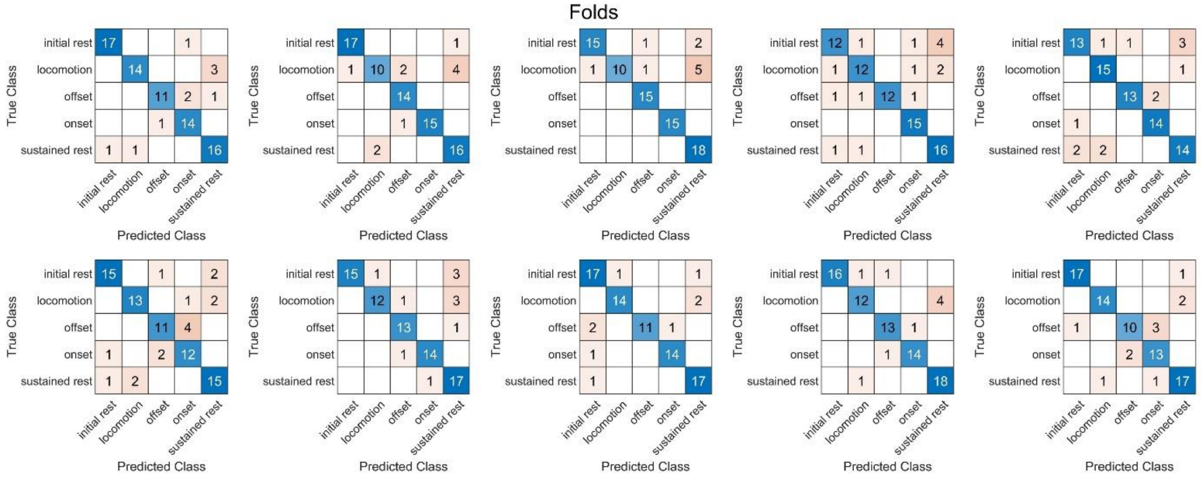


*Supplementary Figure 8. Confusion matrices for each of the 10 outer folds (see above text and methods) for the mice calcium imaging data using logistic regression instead of SVM.*

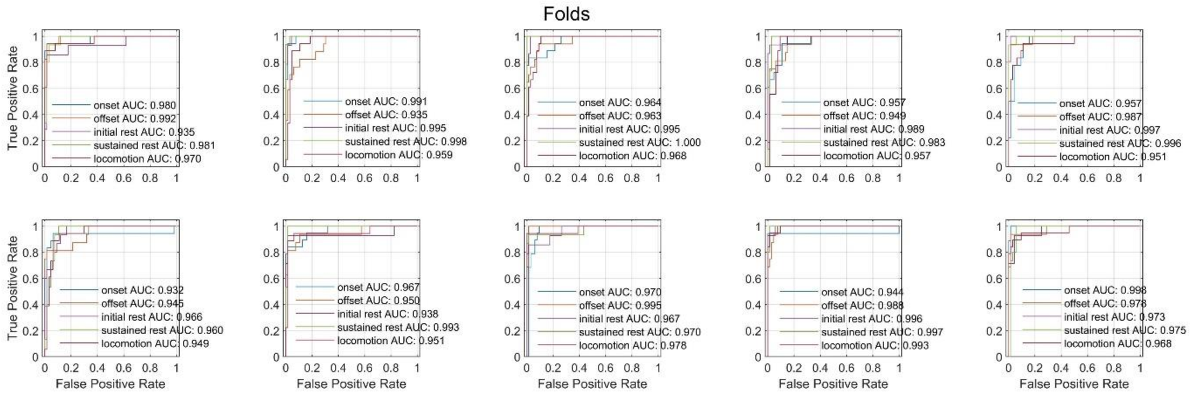


*Supplementary Figure 9. ROC curves for each of the 10 outer folds (see above text and methods) for the mice calcium imaging data using logistic regression instead of SVM. AUC: Area under the curve.*

## *Replication of Rest – Task Difference of INTs Averaged Across Time in Mice Data using ACW – 0*

Supplementary figure 10 shows the comparison of ACW-0 values averaged across windows for each ROI. The difference between mean ACW-0 values for sustained rest and locomotion for all mice is shown in supplementary figure 10A (Wilcoxon test (n=460 for each group, z = 23.05, p < 0.001, r = 0.88). These comparisons for each of the individual mice is shown in supplementary figure 10B (n = 92). In each case, we see an increase in the mean of ACW-0 for each region of interest from sustained rest to locomotion.

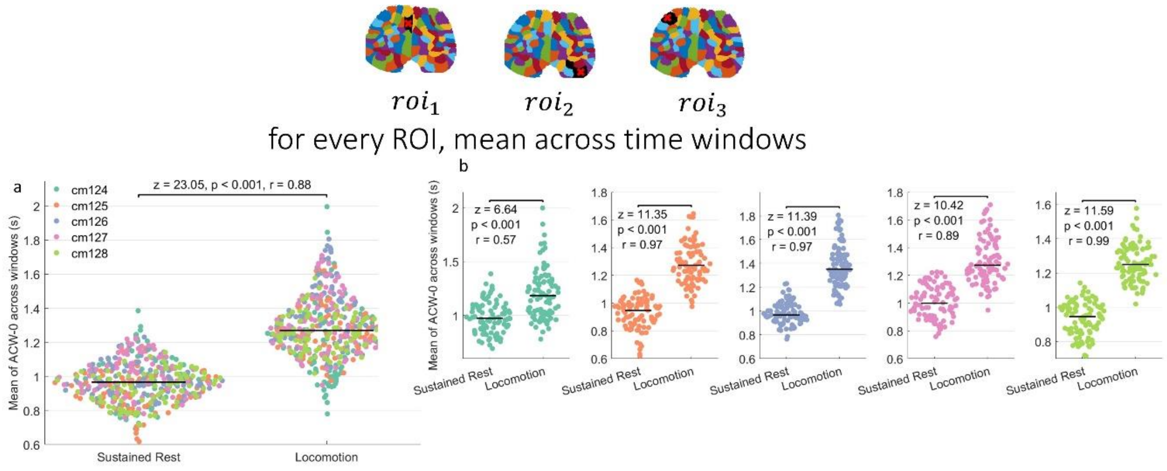


*Supplementary Figure 10. Comparison of mean ACW-0s instead of τs averaged across time for each region of interest (ROI). A. Comparison of ACW-0 values between sustained rest and locomotion states in all mice. B. Comparison for each mouse. Each dot in the figure represents ACW-0 value of one ROI. Colors denote individual mice.*

## Comparison of Mean and Standard Deviation of Neural Activity Across Behavioral States in Mice

Supplementary figure 11 shows the rest – locomotion difference of mean and SD of neural activity in mice data.

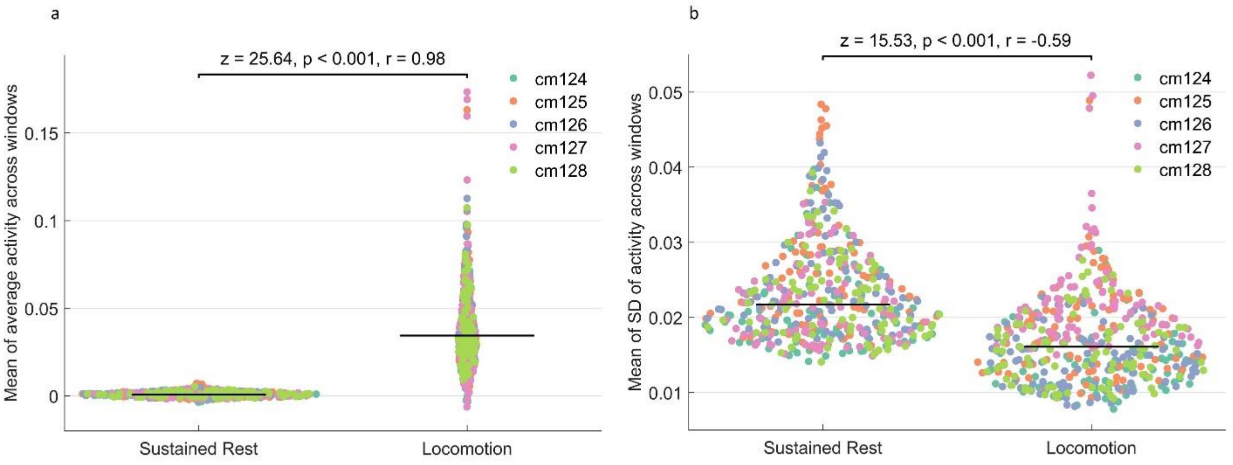


*Supplementary Figure 11. Comparison of mean and SD of neural activity in mice across sustained rest and locomotion conditions.*

## *Additional Control Analyses for the Specificity of INT in Mice Data*

To counter the argument that variance of the data might be a confounding factor for the behavioral specificity of INT, we performed a two – step analysis: first we showed the correlation between variance and INT, second, we used the variance as a confounder in a regression analysis. Supplementary figure 12 shows the correlation whereas supplementary tables 1 and 2 shows the results for logistic regression.

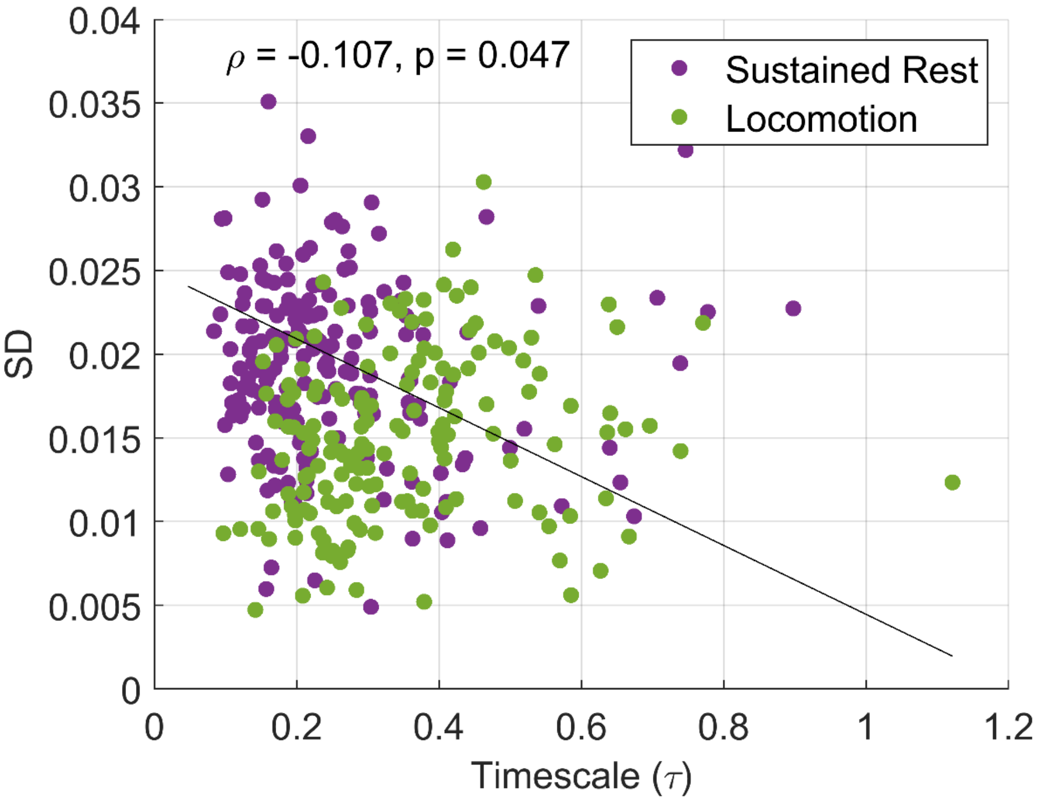


*Supplementary Figure 12. Correlation between ACW and SD in mice data*

*Supplementary table 1. Logistic regression between behavioral state and INT in mice calcium imaging data*

| Name | Estimate | SE | tStat | DoF | p value | 5% CI | 95% CI |
|---|---|---|---|---|---|---|---|
| Intercept | 1.935 | 0.200 | 9.665 | 343 | <0.001 | 1.541 | 2.329 |
| INT | -2.441 | 0.550 | -4.432 | 343 | <0.001 | -3.525 | -1.358 |

*Supplementary table 2. Logistic regression between behavioral state and INT + SD in mice calcium imaging data*

| Name | Estimate | SE | tStat | DoF | p value | 5% CI | 95% CI |
|---|---|---|---|---|---|---|---|
| Intercept | 1.953 | 0.212 | 9.193 | 342 | <0.001 | 1.535 | 2.372 |
| INT | -2.454 | 0.553 | -4.437 | 342 | <0.001 | -3.541 | -1.366 |
| SD | -0.791 | 2.919 | -0.271 | 342 | 0.786 | -6.534 | 4.951 |

## *ROI Specific Rest – Task Comparisons for Mice Data*

We grouped mice ROIs into 12 categories: "Left Frontal", "Left Motor", "Left Somatosensory 1", "Left Somatosensory 2", "Left Somatosensory 3", "Left Visual", "Right Frontal", "Right Motor", "Right Somatosensory 1", "Right Somatosensory 2", "Right Somatosensory 3", "Right Visual". This classification can be seen on a topographic plot in supplementary figure 13. The results for rest – task differences are

presented in supplementary figure 14.

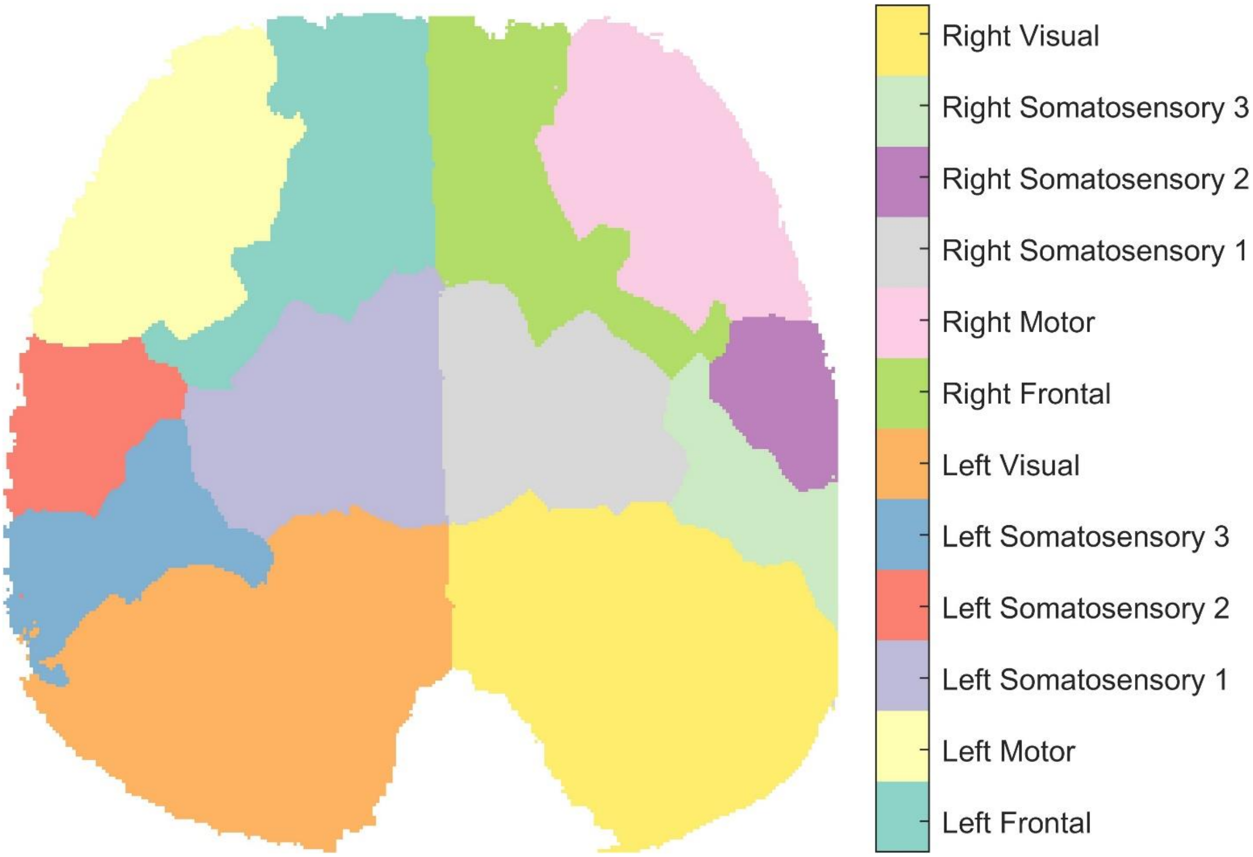


*Supplementary Figure 13. Classification of ROIs in mice calcium imaging data*

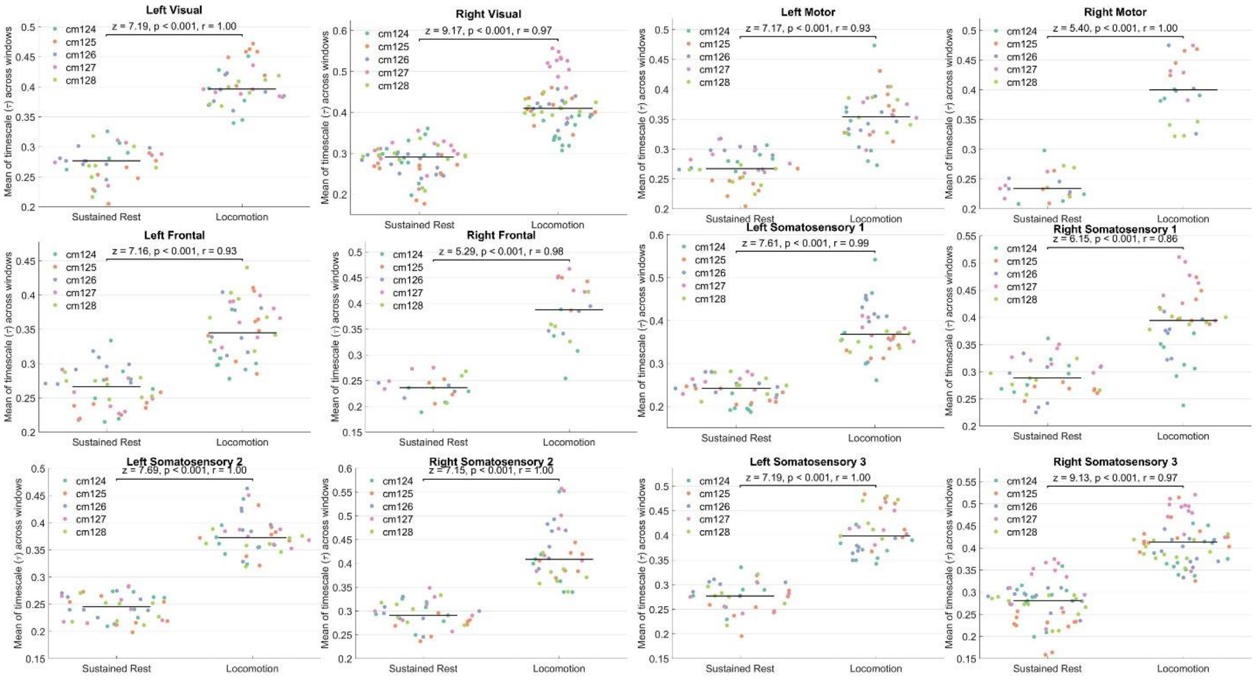


*Supplementary Figure 14. Comparison of τs in mice calcium imaging data according to the classification shown in supplementary figure 6, averaged across time windows for every channel. Every dot denotes one channel. Colors denote mice.*

## Analysis of Early and Late Segments of Mice Data

We compared the early and late periods of sustained rest and locomotion in a recording. We picked recordings that have more than one sustained rest period and compared the first sustained rest and the last one. We did the same analysis for locomotion as well. In both analyses, no significant differences between early and late periods were found (supplementary figure 15).

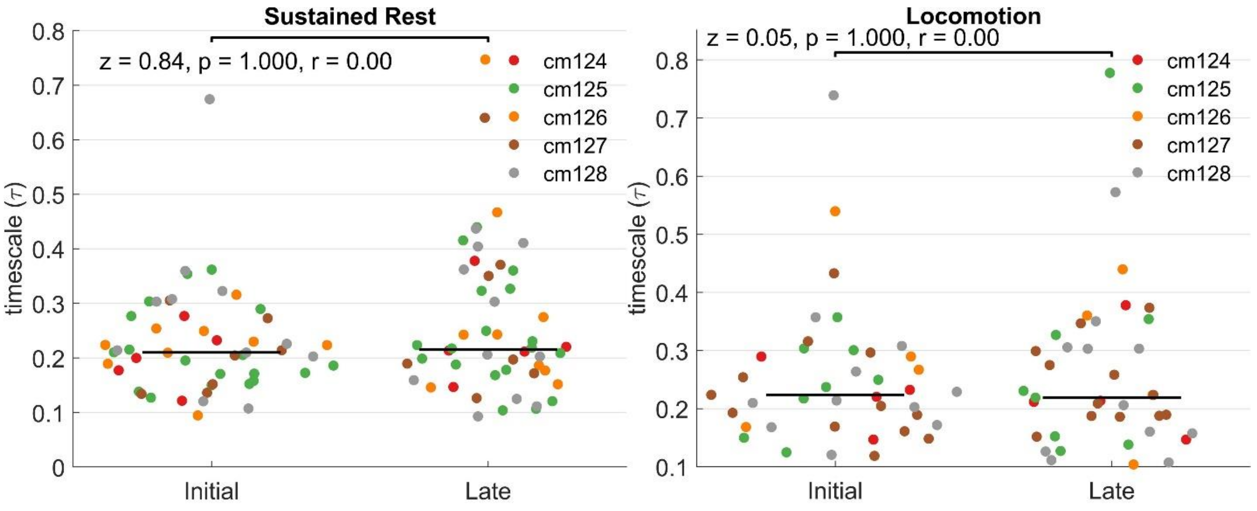


*Supplementary Figure 15. Comparison of early and late periods of Calcium Imaging Data.*

## *Replication of Rest – Task Difference of INTs Averaged Across ROIs in Mice Data using ACW – 0*

Supplementary figure 16 shows the ACW – 0 differences averaged across ROIs for each time window. Supplementary figure 16A shows the change of mean across the brain for each window, showing a significant increase in brain-wide ACW-0 (n=182 for sustained rest, n=163 for locomotion; z = 9.18, p < 0.001, r = 0.57). The comparisons for each of the individual mice are shown in Supplementary figure 16B showing that this increase holds in each mouse (cm124: n=14 for sustained rest, n=15 for cm124, z=2.99, p=0.003, r=0.66; cm125: n=63 for sustained rest, n=36 for locomotion, z=4.67, p<0.001, r = 0.57; cm126: n=26 for sustained rest, n=15 for locomotion, z=3.53, p<0.001, r=0.67; cm127: n=31 for sustained rest, n=48 for locomotion, z=4.13, p<0.001, r=0.55; cm128: n=48 for locomotion, n=49 for sustained rest, z=3.96, p<0.001, r=0.47).

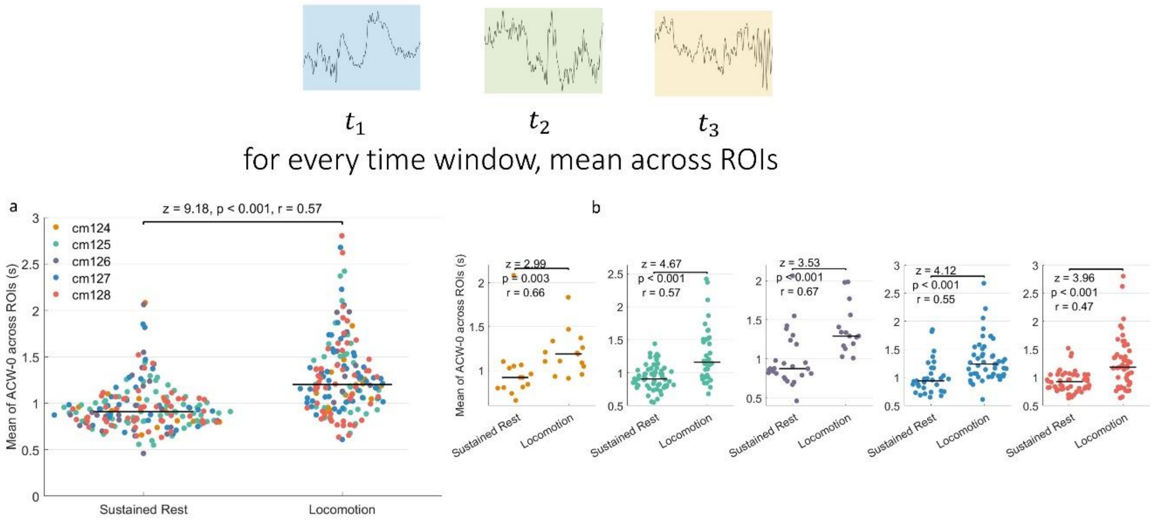


*Supplementary Figure 16. Comparison of mean ACW – 0s instead of τs averaged across regions of interest (ROIs) for each time window. A. Comparison of ACW-0 values between sustained rest and locomotion states in all mice. B. Comparison for each mouse. Each dot in the figure represents ACW-0 value of one time window. Colors denote individual mice.*

## *Analysis of Pupil Diameter in Spontaneous Behavior Task for Mice*

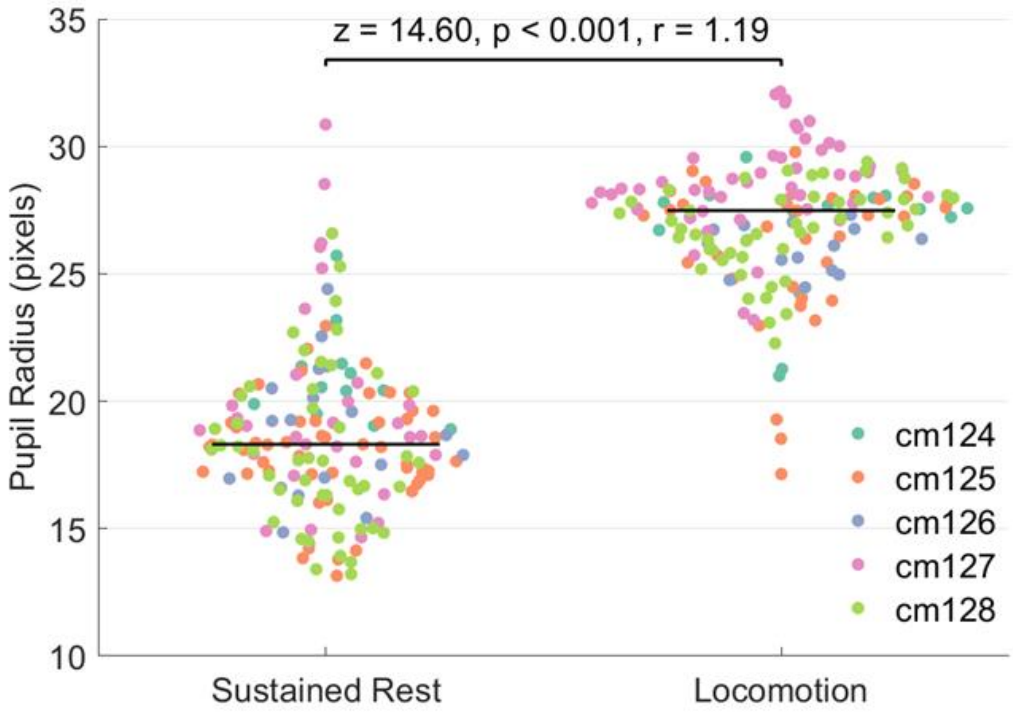


*Supplementary Figure 17. Comparison of pupil size from sustained rest to locomotion. We averaged the recorded pupil size for each time window. Each color denotes one time window.*

We also compared the pupil size between sustained rest and locomotion states to assess the behavioral changes that go along with changes in $\tau$. For each time window, we averaged the pupil size across time. Supplementary figure 17 shows that the pupil size is significantly larger during locomotion compared to the sustained rest (n=182 for sustained rest, n=163 for locomotion; z = 14.6, p < 0.001, r = 1.19).

## *Replication of Rest INT variability – Rest-Task INT Change in Mice Data using ACW – 0*

In supplementary figure 18, we present the correlations between rest variability of ACW – 0 and percent rest – task change of it (figure 18A: Spearman's $\rho(458)$ = -0.44, p < 0.001). Supplementary figure 18B shows the distribution of rest variability and the rest-locomotion percent change across the brain. Supplementary figure 18C shows this relationship for each of the mice (n=92 in each plot), showing that

all mice show this negative and significant correlation.

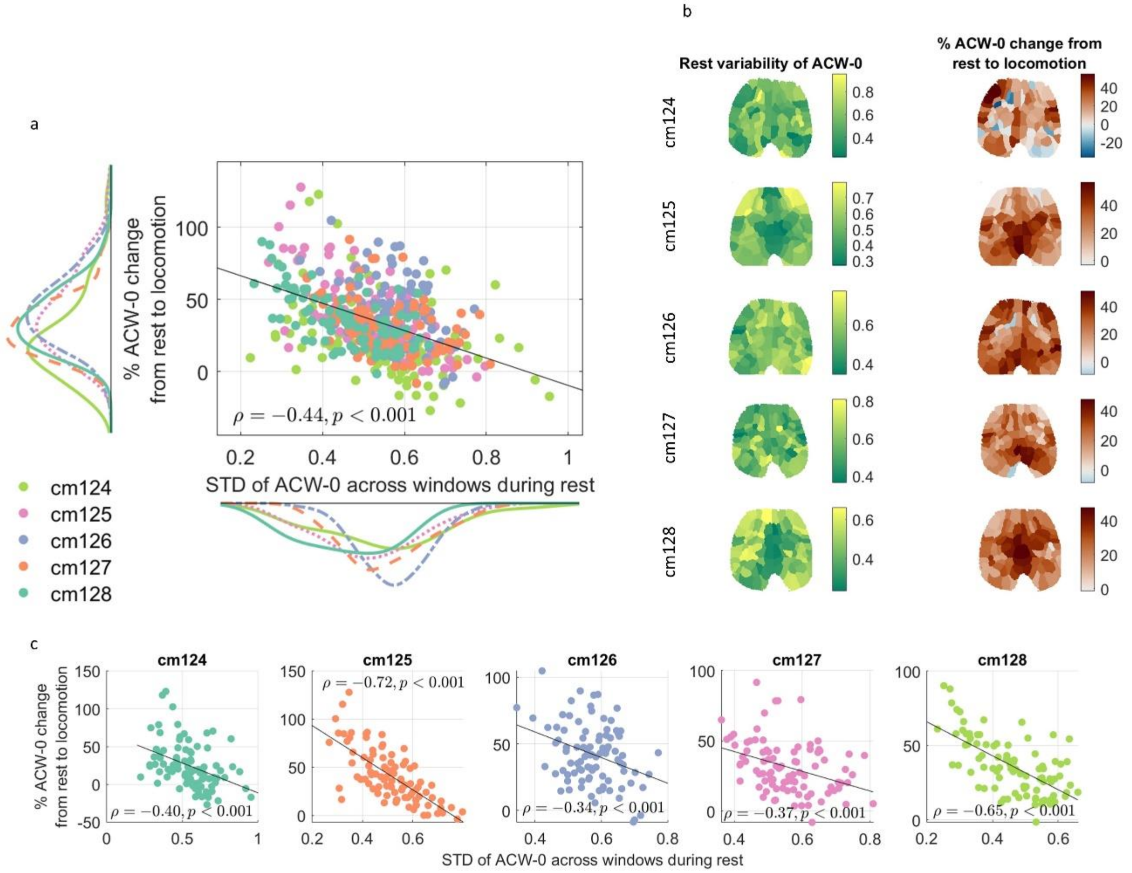


*Supplementary Figure 18. Rest-Behavior modulation of ACW – 0 instead of τ. A. We calculated variability of ACW-0s across time windows. To calculate the change of ACW-0s from rest to locomotion, we averaged the percent change of ACW-0s in each of the aforementioned windows. We correlated variability of ACW-0s during sustained rest with the ACW-0 percent change from rest to locomotion. Each dot denotes one region of interest in one recording session. B. The distribution of variability of ACW-0 during rest and percent change of ACW-0 from rest to locomotion across the brain. C. Same as panel A for each of the individual mice.*

## ROC Curves and Confusion Matrices for Each Fold for EEG

Here, we report the confusion matrices and ROC curves for each of the tests on 10 outer folds for the mice data (supplementary figures 19 and 20).

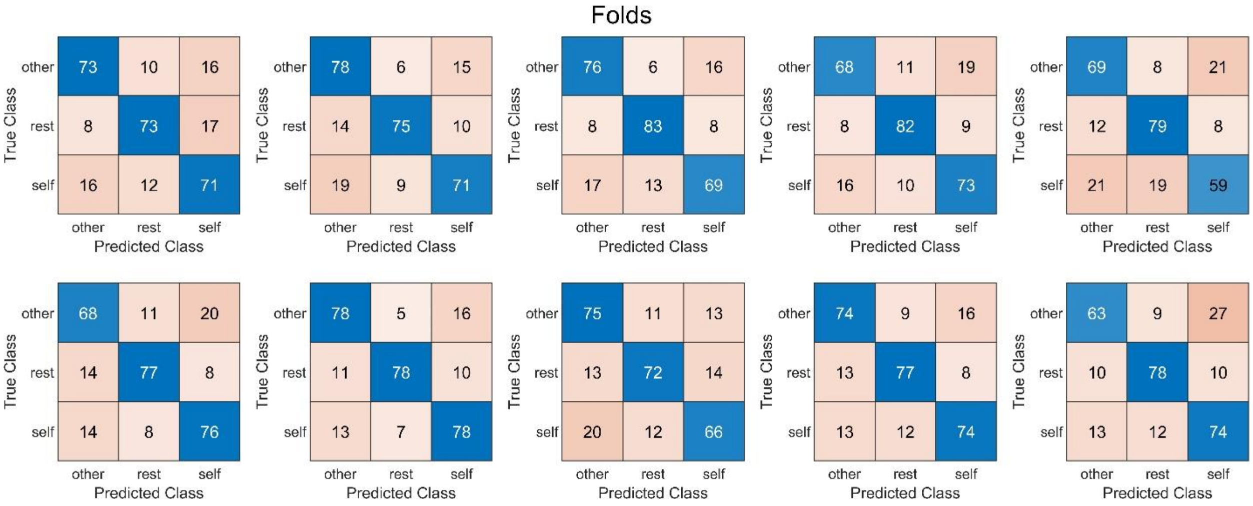


*Supplementary Figure 19. Confusion matrices for each of the 10 outer folds (see above text and methods) for the human EEG data.*

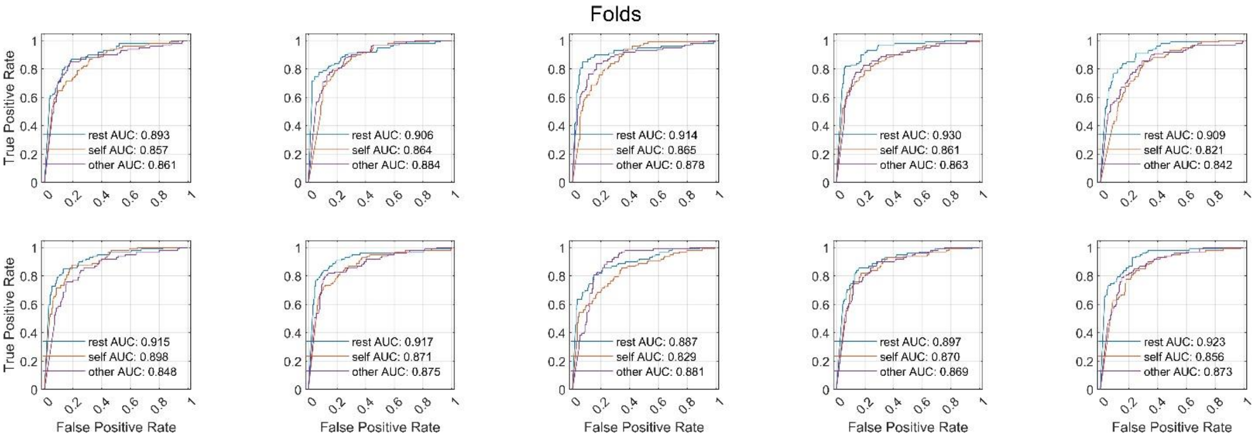


*Supplementary Figure 20. ROC curves for each of the 10 outer folds (see above text and methods) for the human EEG data. AUC: Area under the curve.*

## Replication of ROC Curves and Confusion Matrices in EEG Data using ACW – 0

Supplementary figure 21A shows the distribution of ACW-0 values across the scalp averaged across time windows and 21 subjects (2961 time windows and 64 channels as features). We started our analysis by replicating the support vector machine (SVM) findings with a radial basis kernel. The SVMs were trained using nested cross validation with 10 inner and 10 outer folds. The confusion matrix for test data aggregated across folds is given in supplementary figure 16B, showing that the SVM is reaching an accuracy of at least 58.3% while random chance is 33.3%. ROC curves averaged across folds are given in

supplementary figure 16C, showing a minimum AUC of 0.76, indicating that indeed the different states (rest, self, non-self) can be distinguished by ACW-0 values recorded on scalp. Confusion matrices and ROC curves for individual folds can be found in supplementary figures 22 and 23.

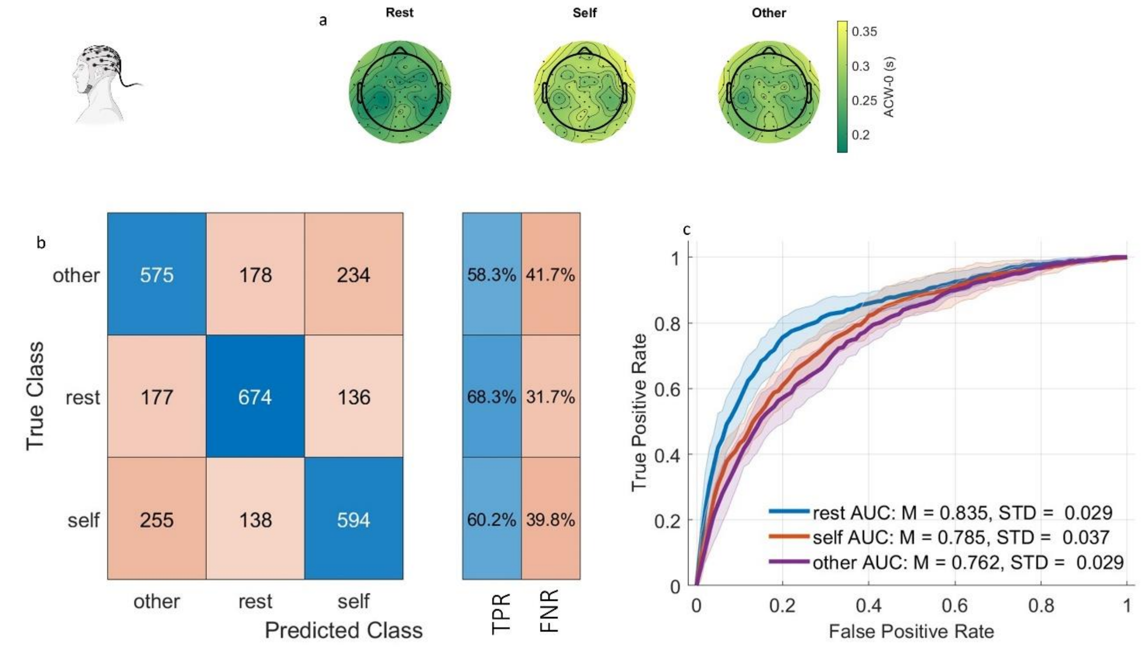


*Supplementary Figure 21. Classification learning for EEG states. A. The distribution of ACW-0 values averaged across time windows and subjects on the scalp for rest, self-narrative and non-self (other) narrative. B. We trained support vector machines to classify rest versus self versus other states using ACW-0 values across the brain using nested cross validation for hyperparameter tuning (10 inner, 10 outer folds). Panel B shows the confusion matrix for test data, aggregated across 10 folds. C. Receiver operating characteristic curve for the test data of the trained support vector machine. The ROC curves for 10 folds were averaged and the standard deviation across folds was indicated with the shadings. Abbreviations: TPR: True positive rate, FNR: False negative rate, AUC: Area under the curve, M: Mean, STD: Standard deviation.*

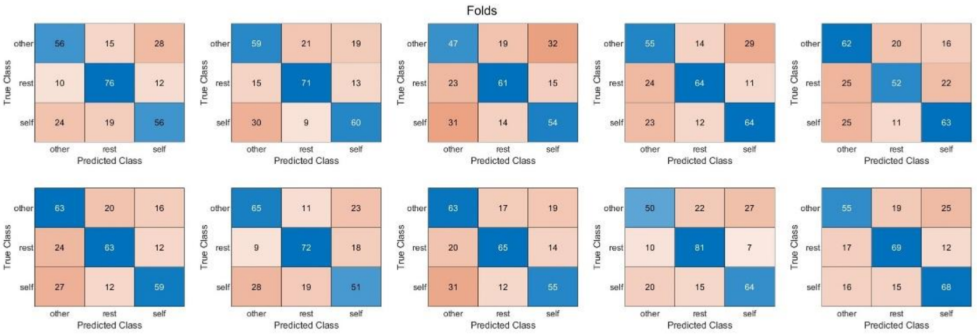


*Supplementary Figure 22. Confusion matrices for each of the 10 outer folds (see above text and methods) for the human EEG data using ACW – 0 instead of $\tau$.*

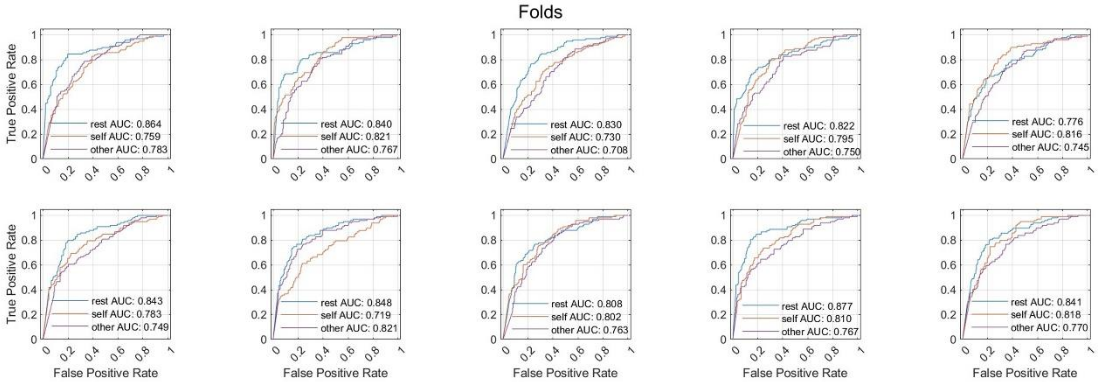


*Supplementary Figure 23. ROC curves for each of the 10 outer folds (see above text and methods) for the human EEG data using ACW – 0 instead of $\tau$. AUC: Area under the curve.*

## Logistic Regression Results for EEG Data

As explained above for the case of mice, we additionally did logistic regression for EEG data as well. Supplementary figure 24 shows the average ROC and aggregate confusion matrix. Supplementary figures 25 and 26 show confusion matrices and ROC curves for each fold.

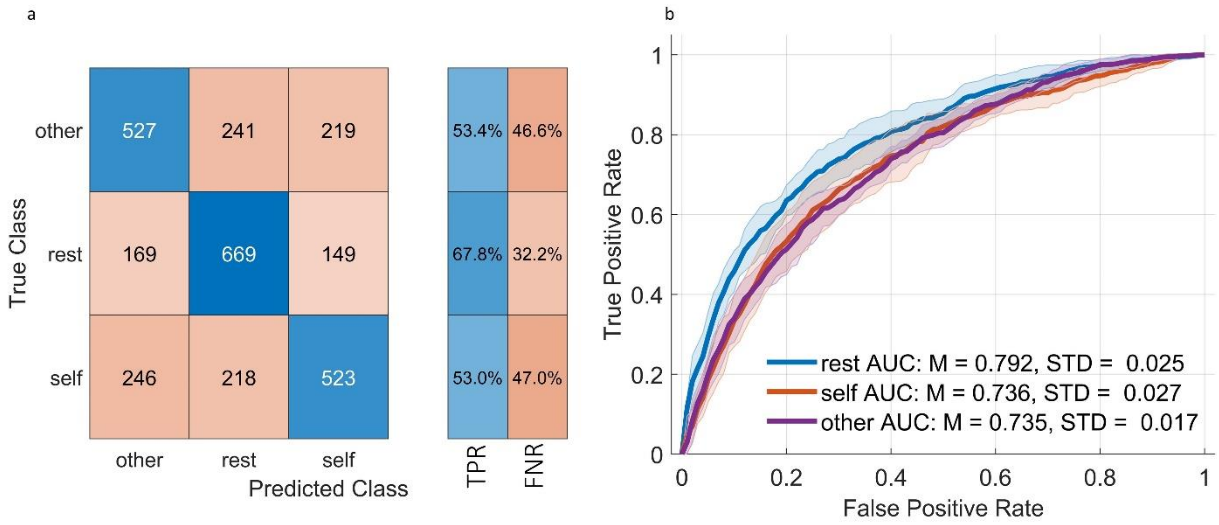


*Supplementary Figure 24. Classification learning for behavioral states using logistic regression instead of SVM in human EEG data. a. We trained logistic regression models to classify the behavioral states of mice based on the ACW-0 values across the brain with nested cross validation for hyperparameter tuning (10 inner, 10 outer folds). Panel a shows the confusion matrix for test data, aggregated across folds. b. Receiver operating characteristic curve for the test data using the logistic regression model. The data ROC curves from 10 folds were averaged and the standard deviation across the folds were indicated as shading. Abbreviations: TPR: True positive rate, FNR: False negative rate, AUC: Area under the curve, M: Mean, STD: Standard deviation*

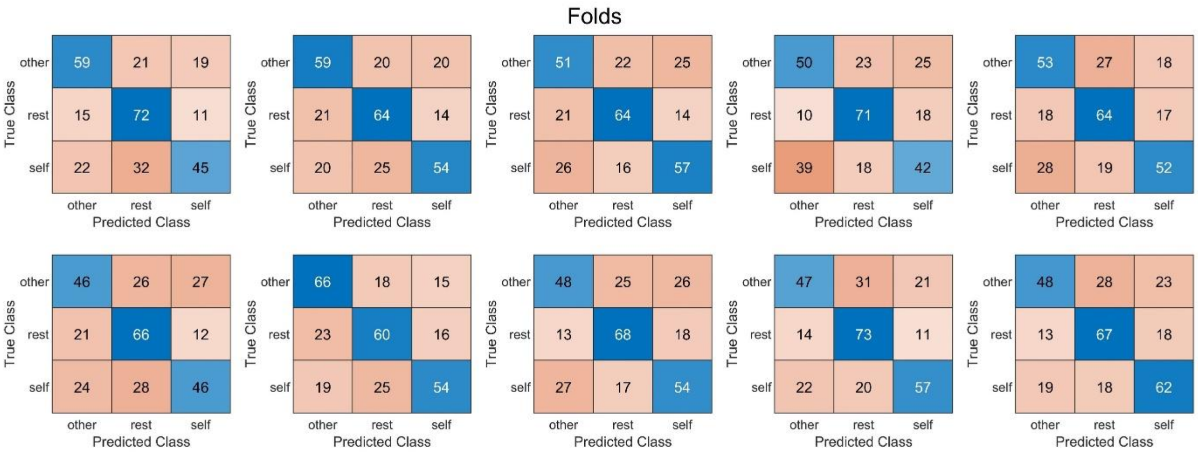


*Supplementary Figure 25. Confusion matrices for each of the 10 outer folds (see above text and methods) for the human EEG data using logistic regression instead of SVM.*

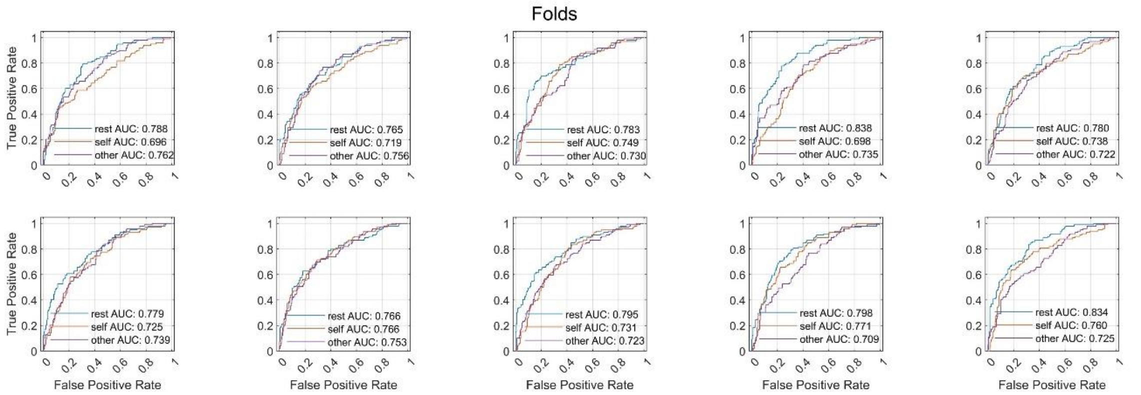


*Supplementary Figure 26. ROC curves for each of the 10 outer folds (see above text and methods) for the human EEG data using logistic regression instead of SVM. AUC: Area under the curve.*

## *Rest Variability – Rest-Task Change Correlations in EEG data*

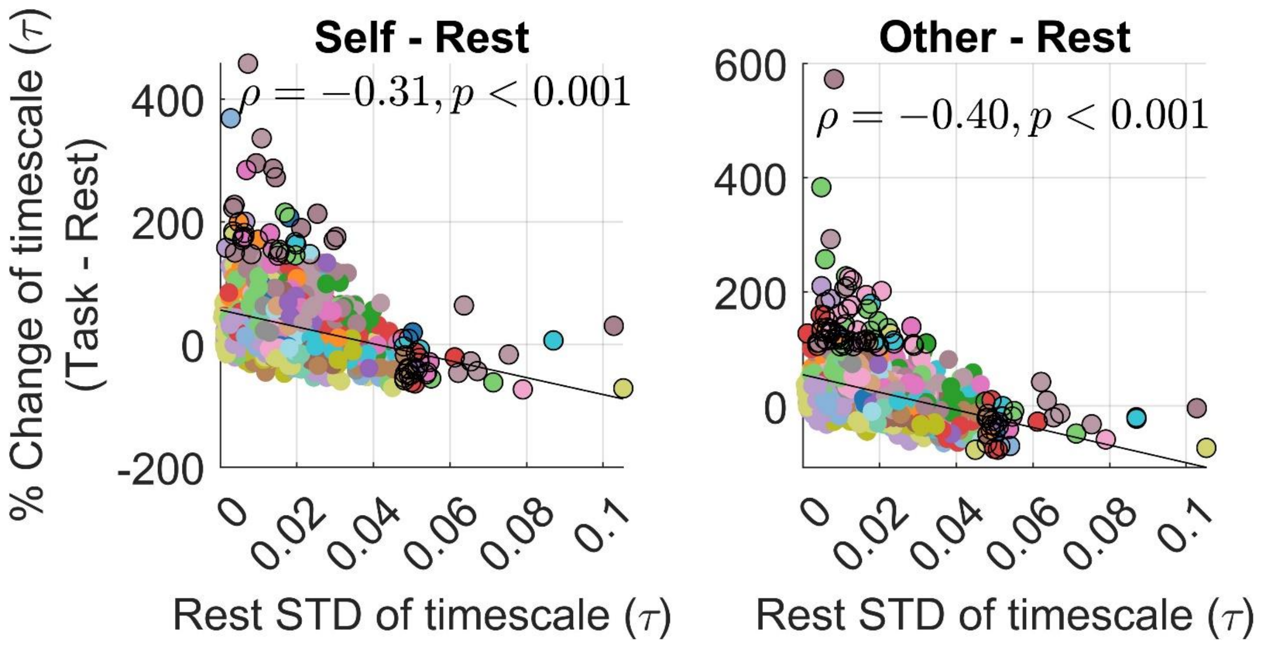


*Supplementary Figure 27. We calculated the variability across time windows for each channel during the resting state and correlated that with the percent rest – task change of $\tau$. Each dot denotes one channel, colors denote subjects. The dots with black circles around them are outliers taken out from figure 7D.*

Supplementary figure 27 shows the Spearman correlation between rest variability of $\tau$ and rest-task % change of it (rest-self difference: Spearman's $\rho(1342) = -0.31$, p<0.001; rest-other difference: Spearman's $\rho(1342) = -0.4$, p<0.001).

## *Replication of Rest – Task INT Comparisons and Correlations using ACW – 0*

Supplementary figure 28A shows that during the self-stimulus, INT are the longest, followed by other and rest (n=1344 for each group). Multiple comparisons using Wilcoxon tests and effect size calculations can be seen in Supplementary figure 28 panels A and B. A mixed effects model treating average ACW – 0 across time windows as dependent variable, state as fixed effect, channels and subjects as random effects show significant effect for state ($F_{2,\ 3946}$=288.396, p < 0.001). Similarly, mixed effects model treating average ACW - 0 across channels as dependent variable, state as fixed effect and subjects as random effect found significant effect for state ($F_{2,\ 2938}$ = 99.193, p < 0.001).

As in the mice, we also looked at the rest variability of INT and rest-behavior change of the mean of INT. Supplementary figure 20C shows resting state variability of ACW-0 values as well as rest-behavior changes of the mean of ACW-0s. In figure 28D, we see the correlation between resting variability and rest-behavior percent change of ACW-0 values (rest-self difference: Spearman's $\rho(1342) = -0.21$, p < 0.001; rest-other difference: $\rho(1342) = -0.24$, p < 0.001).

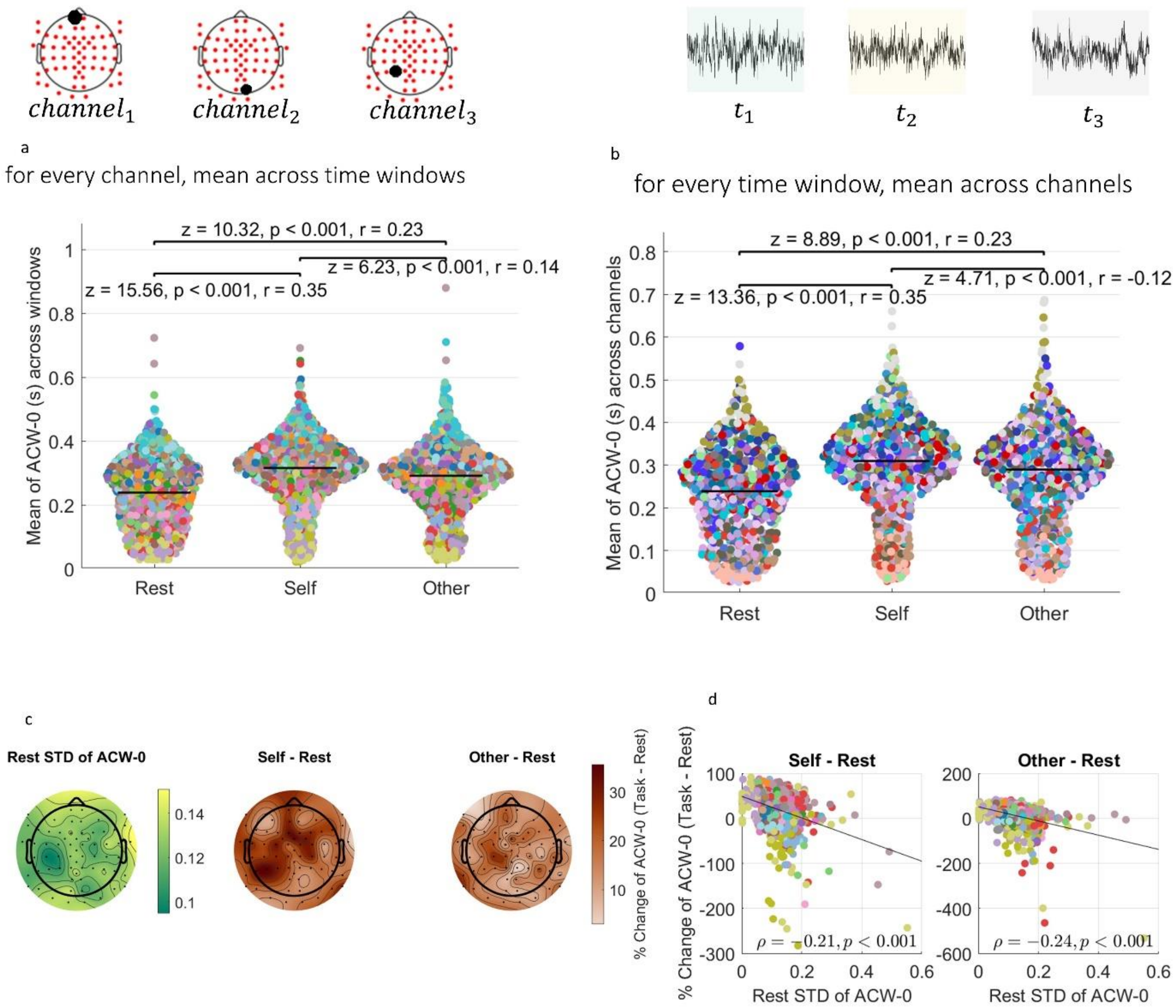


*Supplementary Figure 28. A. Comparison of ACWs between three states averaged across time windows for every channel. Every dot denotes one channel. Colors denote subjects. B. Comparison of ACWs across three states, averaged across channels for every time window. Every dot denotes one time window. Colors denote subjects. C. Resting state variability and rest-task change of ACWs averaged across time windows and subjects. D. We calculated the variability across time windows for each channel during the resting state and correlated with the percent rest – task change of ACW-0. Each dot denotes one channel, colors denote subjects.*

## Comparison of Power Bands in Human EEG Data

In addition to timescales, we also calculated the power bands in human EEG data and compared them across the three conditions. Supplementary figures 29 and 30 show the results and results without outliers, defined as the values which are three median absolute deviations away from the median.

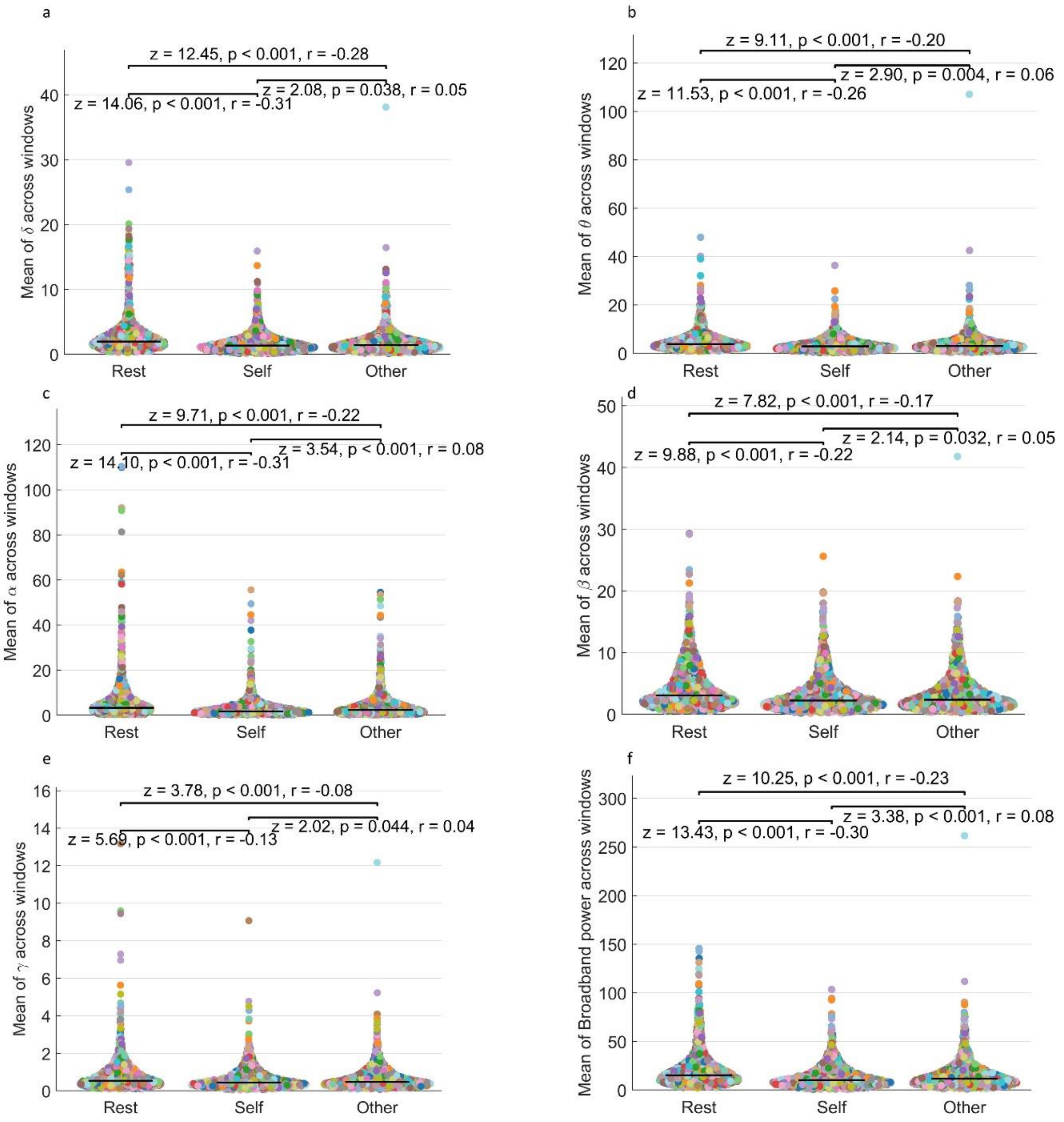


*Supplementary Figure 29. Comparison of power bands across three states in human EEG data. Horizontal black lines show median.*

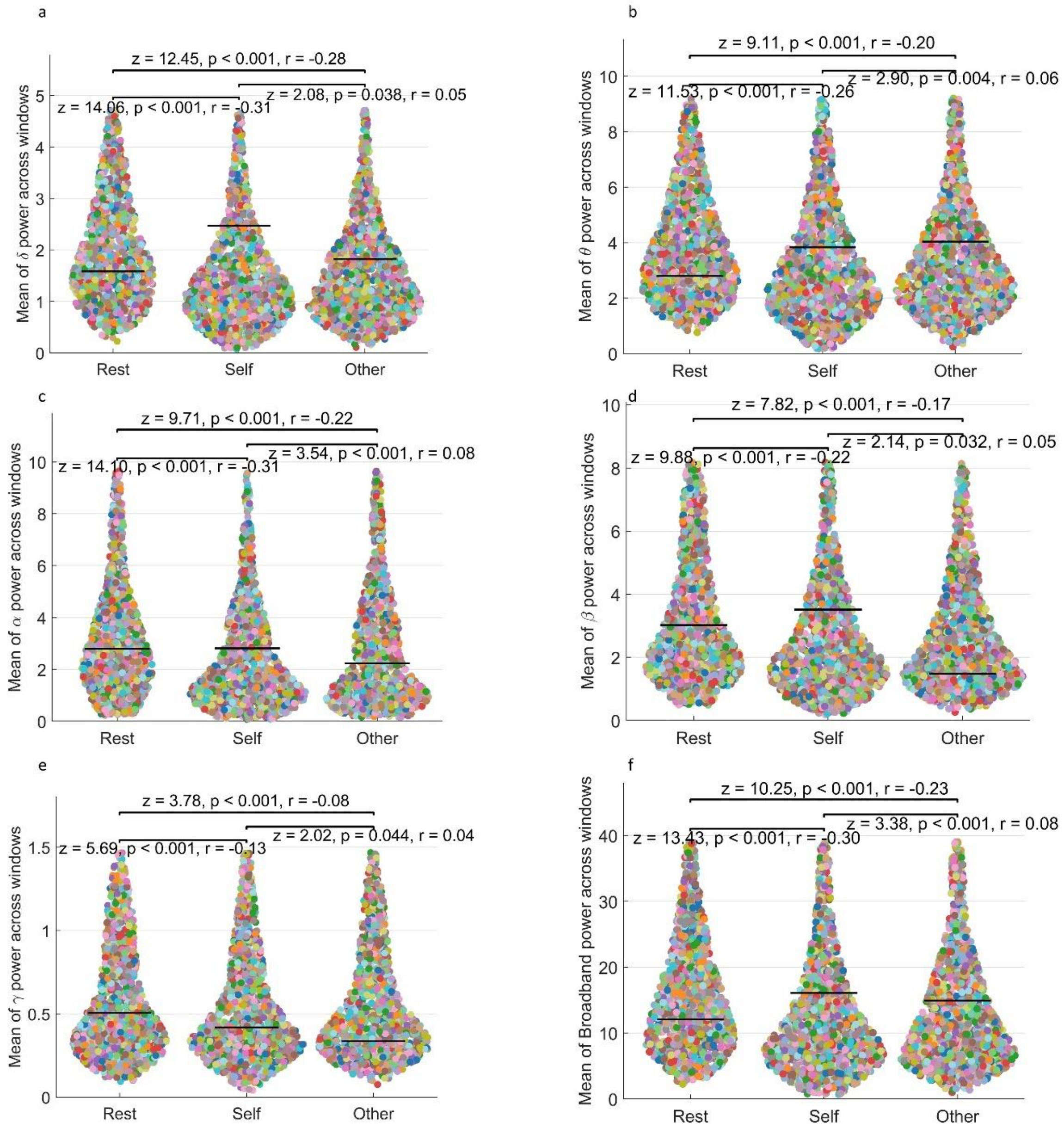


*Supplementary Figure 30. Comparison of power bands across three states in human EEG data without the outliers which are defined as three median absolute deviations away from the median. Horizontal black lines show median.*

## *Additional Control Analyses for the Specificity of INT in EEG Data*

To counter the argument that the power bands in EEG data might be a confounding factor for the behavioral specificity of INT, we performed a two – step analysis: first we showed the correlation between power bands and INT, second, we used the power bands as a confounder in a regression analysis. Supplementary figure 31 shows the correlation whereas supplementary tables 3 and 4 shows the results for logistic regression.

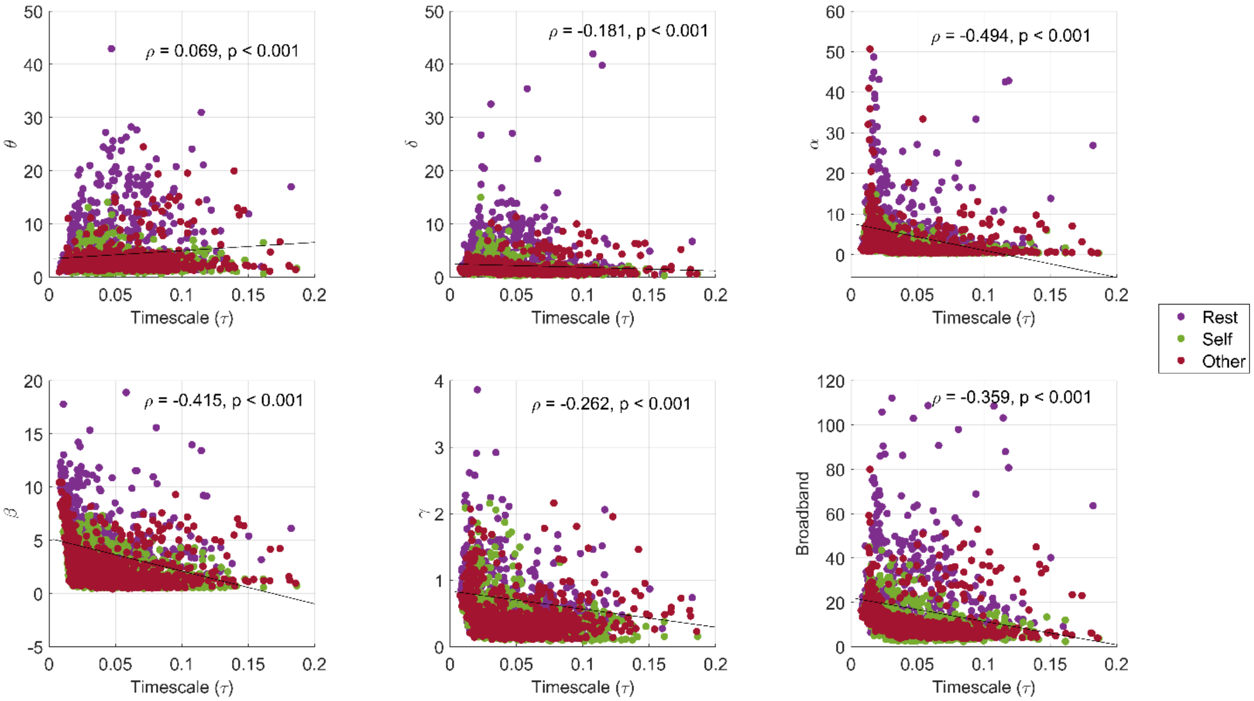


*Supplementary Figure 31. Correlation between oscillatory power bands and INT.*

*Supplementary table 3. Logistic regression between behavioral state and INT in human EEG data*

| Name | Estimate | SE | tStat | DoF | p value | 5% CI | 95% CI |
|---|---|---|---|---|---|---|---|
| Intercept | 0.664 | 0.007 | 86.03 | 189500 | 0 | 0.649 | 0.679 |
| INT | 0.637 | 0.152 | 4.171 | 189500 | <0.001 | 0.338 | 0.936 |

*Supplementary table 4. Logistic regression between behavioral state and INT + power bands in human EEG data*

| Name | Estimate | SE | tStat | DoF | p value | 5% CI | 95% CI |
|---|---|---|---|---|---|---|---|
| Intercept | 0.724 | 0.014 | 50.556 | 189500 | 0 | 0.696 | 0.752 |
| INT | 0.471 | 0.170 | 2.760 | 189500 | 0.005 | 0.136 | 0.806 |
| Delta | -0.012 | 0.002 | -5.963 | 189500 | <0.001 | -0.017 | -0.008 |
| Theta | 0.013 | 0.003 | 4.139 | 189500 | <0.001 | 0.006 | 0.019 |
| Alpha | -0.007 | 0.001 | -4.063 | 189500 | <0.001 | -0.010 | -0.003 |
| Beta | -0.003 | 0.003 | -1.296 | 189500 | 0.195 | -0.009 | 0.002 |
| Gamma | 0.022 | 0.018 | 1.247 | 189500 | 0.212 | -0.012 | 0.058 |
| Broadband | 0.001 | 0.001 | 0.083 | 189500 | 0.933 | -0.003 | 0.003 |

## *Channel – Specific Analyses for EEG Data*

We performed a topographic analysis in EEG data by classifying channels in following categories: 'Left Frontal', 'Right Frontal', 'Left Central', 'Right Central', 'Left Parietal', 'Right Parietal', 'Left Temporal', 'Right Temporal', 'Left Occipital', and 'Right Occipital'. This classification can be seen on a topographic plot in supplementary figure 32.

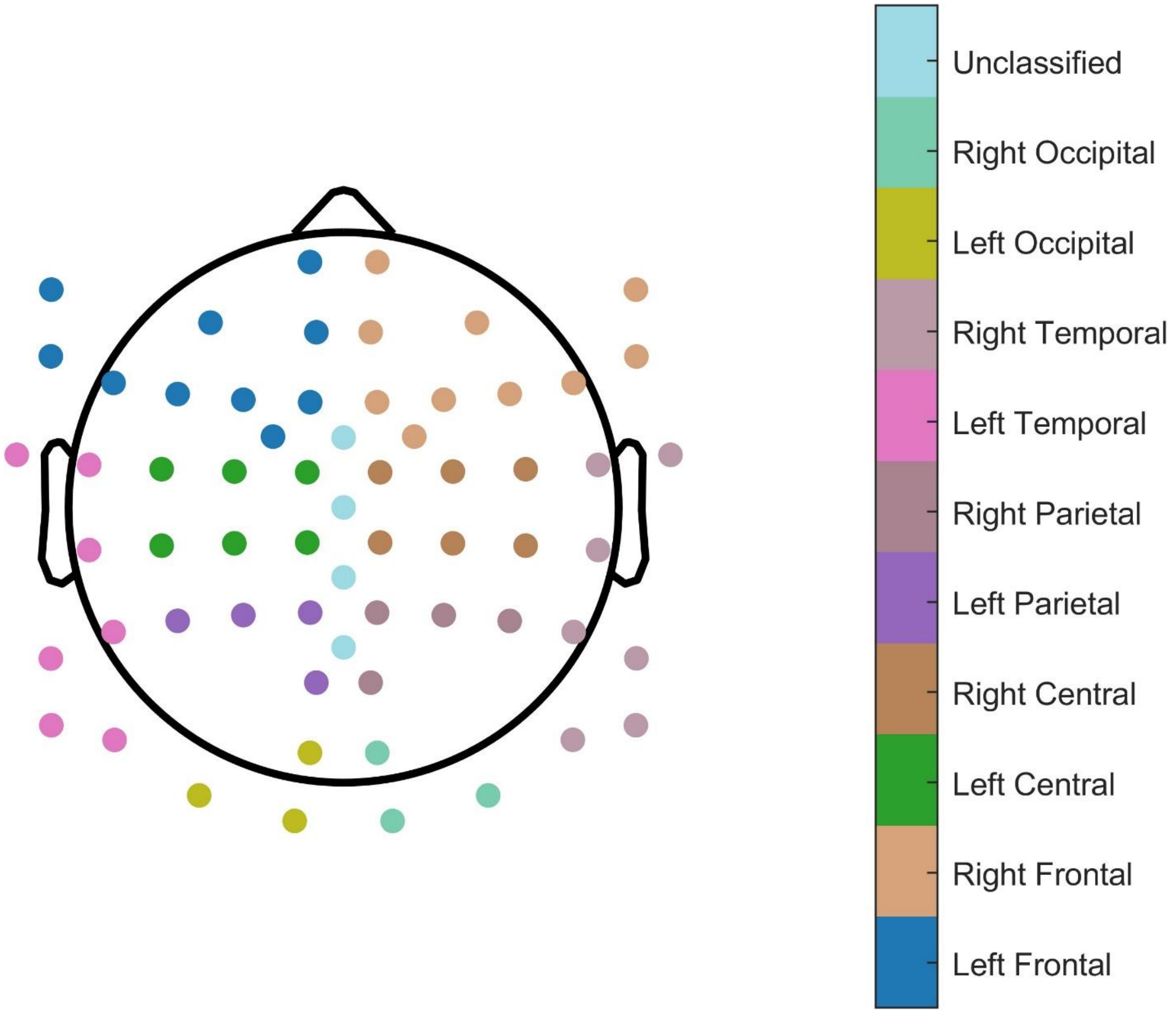


*Supplementary Figure 32. Classification of electrodes based on locations on scalp*

We performed rest – task difference analyses on individual groups of channels. These results are visualized in supplementary figure 33.

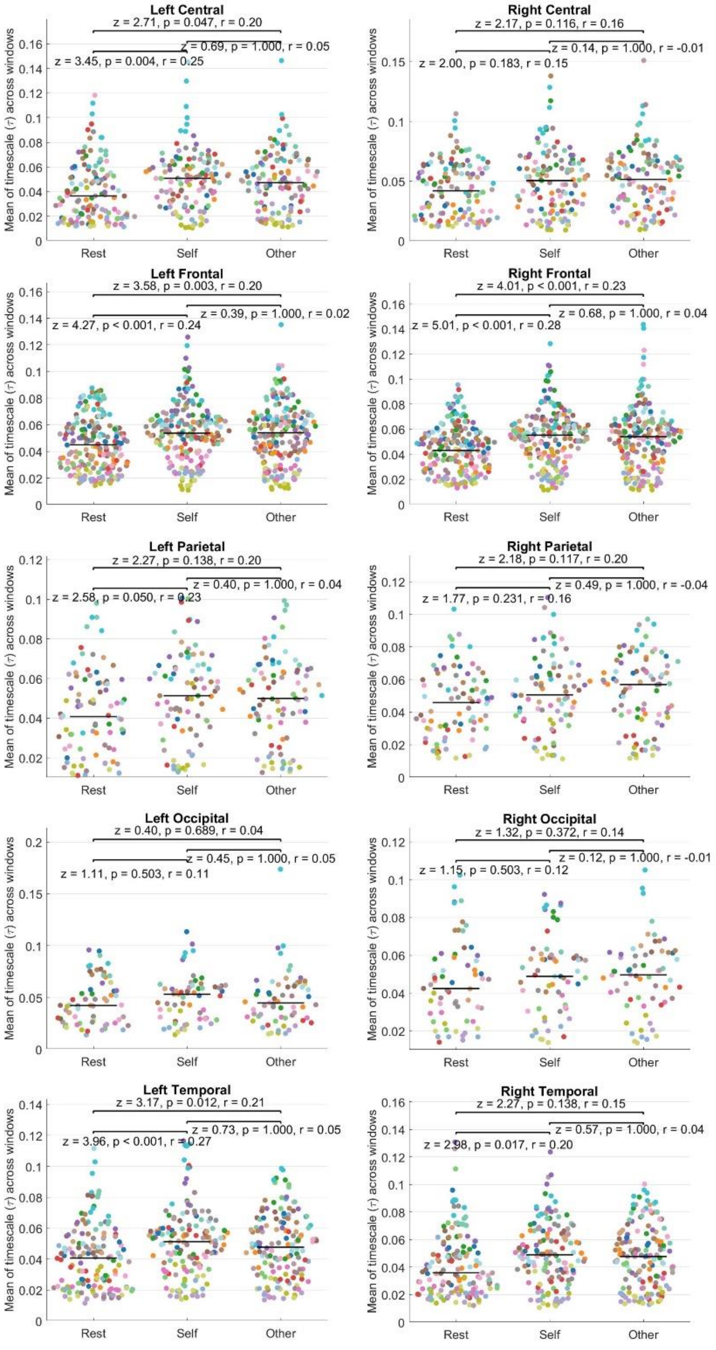


*Supplementary Figure 33. Comparison of τs in human EEG between three states, rest and two task states, according to the classification shown in supplementary figure 9, averaged across time windows for every channel. Every dot denotes one channel. Colors denote subjects.*

## Replication of Modeling Results using ACW – 0

We proceeded with the modeling results. Supplementary figure 34 shows comparison of ACW – 0 values between rest and stimulated states (panel A, n=10800 for each group, z=50.68, p<0.001, r=0.40) as well

as the rest variability – rest-task percent change correlation for ACW – 0 (panel B, $\rho(10798)=-0.521$, $p<0.001$).

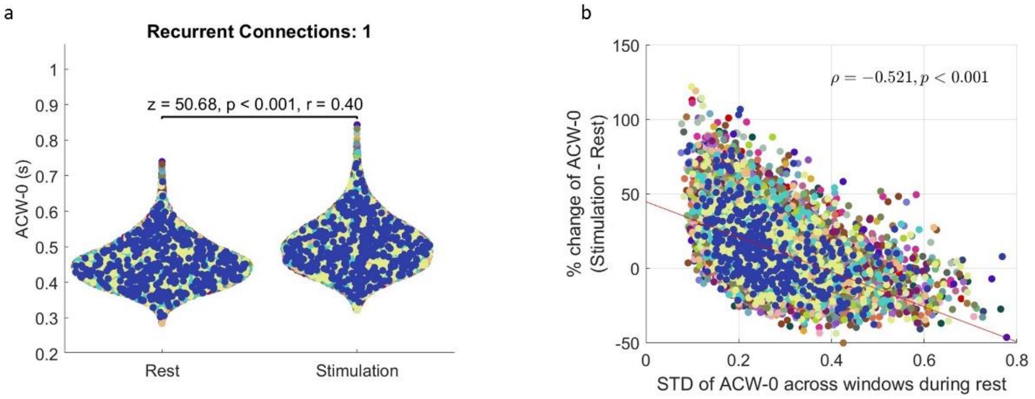


*Supplementary Figure 34. Replication of empirical results in a neural mass model using ACW – 0 instead of τ. A. We simulate the model for 300 seconds, discard the first 100 seconds and calculate ACW-0 values in 10 second sliding windows with no overlap in both rest and stimulated states. For every region, we averaged the ACW-0 values across time and compared between rest and stimulated conditions. B. We calculated the variability of ACW-0 values across time for every region and correlated it with the percent change of ACW-0 from rest to stimulated state. In panels D and E, each dot represents one region. Colors denote simulations.*

Supplementary figure 35 shows rest – task change of ACW – 0 for different values of recurrent connections. Figure 35B shows that for recurrent connections lower than the default strength of connection (1), the rest-stimulated state change of ACW-0 is lower with negligible difference in the absence of recurrent connections (n=10800 for each group in all comparisons; z = 24.78, p < 0.001, r = 0.19 for $W_{ii}$=0.5 and z = 5.93, p < 0.001, r = 0.05 for $W_{ii}$=0). For higher recurrent connections, the difference initially increases, then stops (z = 85.87, p < 0.001, r = 0.67 for $W_{ii}$=1.5; z = 114.85, p < 0.001, r = 0.9 for $W_{ii}$=2). Increasing the recurrent connections further causes a decrease in the rest-stimulated state change with negligible difference at the connection strength 3 (z = 81.94, p < 0.001, r = 0.64 for $W_{ii}$=2.5 and z = 6.30, p < 0.001, r = 0.05 for $W_{ii}$=3). Increasing even further causes a decrease in the ACW-0 (z = 69.30, p < 0.001, r = -0.54 for $W_{ii}$=3.5; z = 112.39, p < 0.001, r = -0.88 for $W_{ii}$=4). These results are summarized in figure 35A. We plotted the changes averaged across ROIs and simulations and interpolated the values between our simulations to obtain the continuous line.

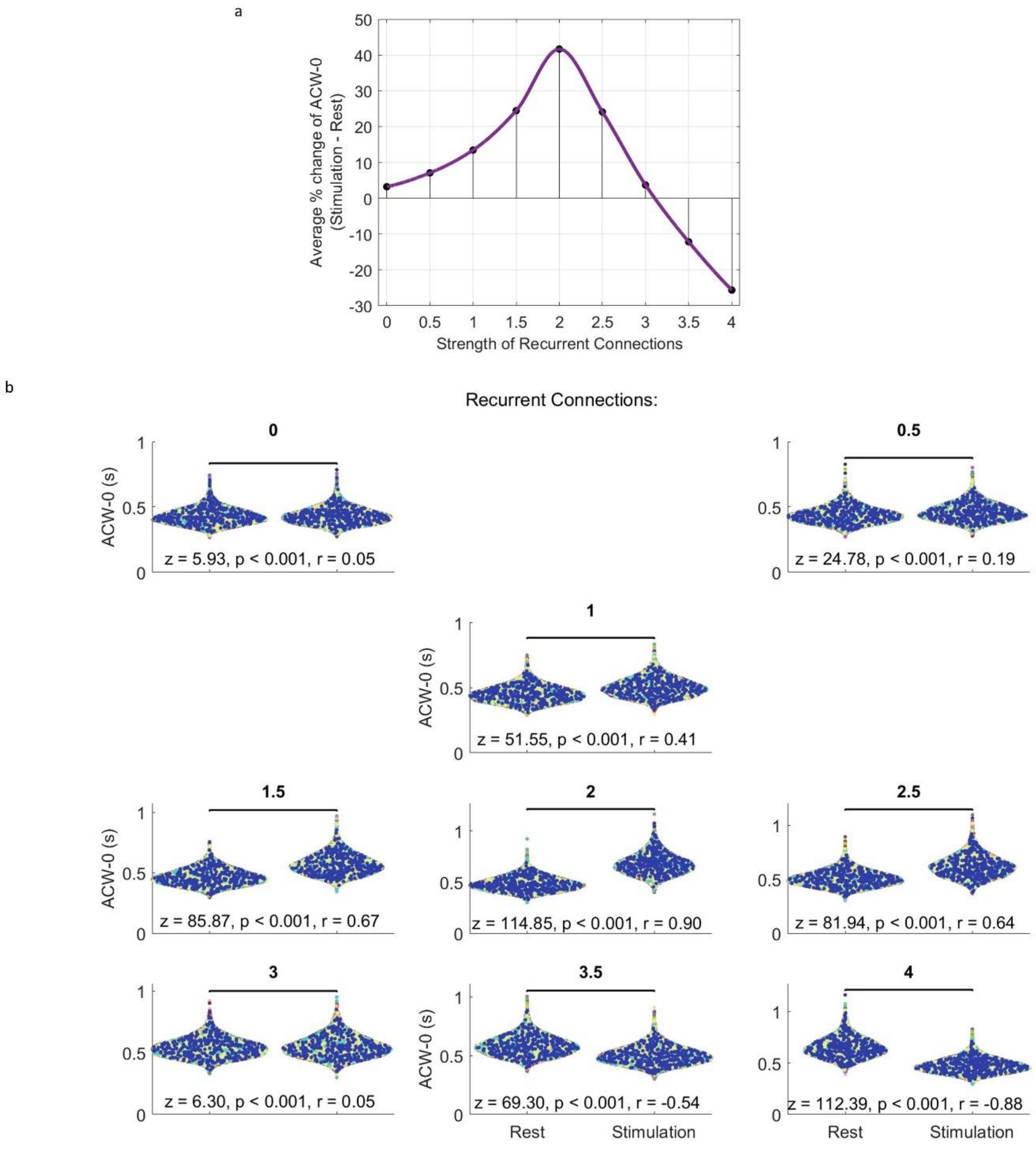


*Supplementary figure 35. Recurrent connections determine rest – stimulation ACW-0 change. A. Relationship between recurrent connections and rest-stimulation change of ACW-0. The positions of the black dots are calculated as the percent change of ACW-0 averaged across regions and simulations. Continuous line was interpolated from the dots. B. We simulate the models with various strengths of recurrent connections and compare the ACW-0 values in rest and stimulated states. As in figure 9D, each dot denote one region and colors denote simulations.*

## Controlling for Firing Rates in the Neural Mass Model

It can be argued that the findings presented in figure 9 depend on the firing rate instead of recurrent connections. Indeed when all else is equal, increasing the strength of recurrent connections increase the firing rate. This can be observed in supplementary figure 36 for resting state (no stimulation) and supplementary figure 37 for stimulated state.

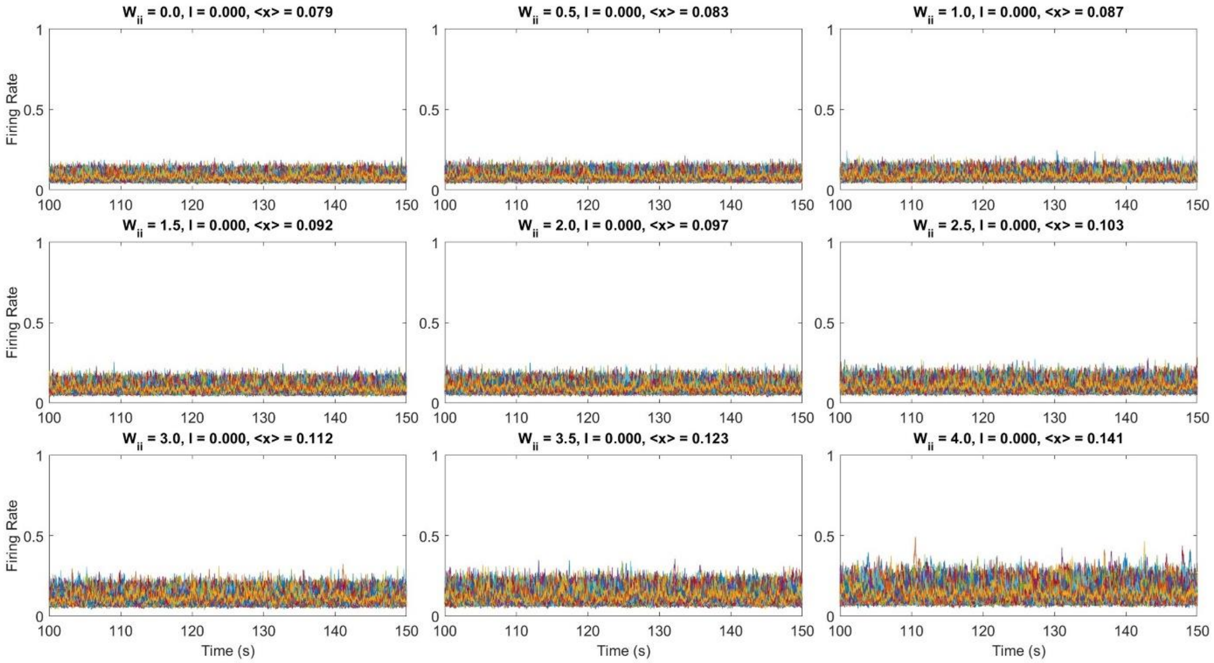


*Supplementary Figure 36: Firing rate time series in one simulation for all regions in resting state.* $W_{ii}$ *denote recurrent connections.* $I$ *stands for external input (which is set to 0 for the resting state) and* $< x >$ *is the average firing rate across time and regions of interest. Each color denotes one region.*

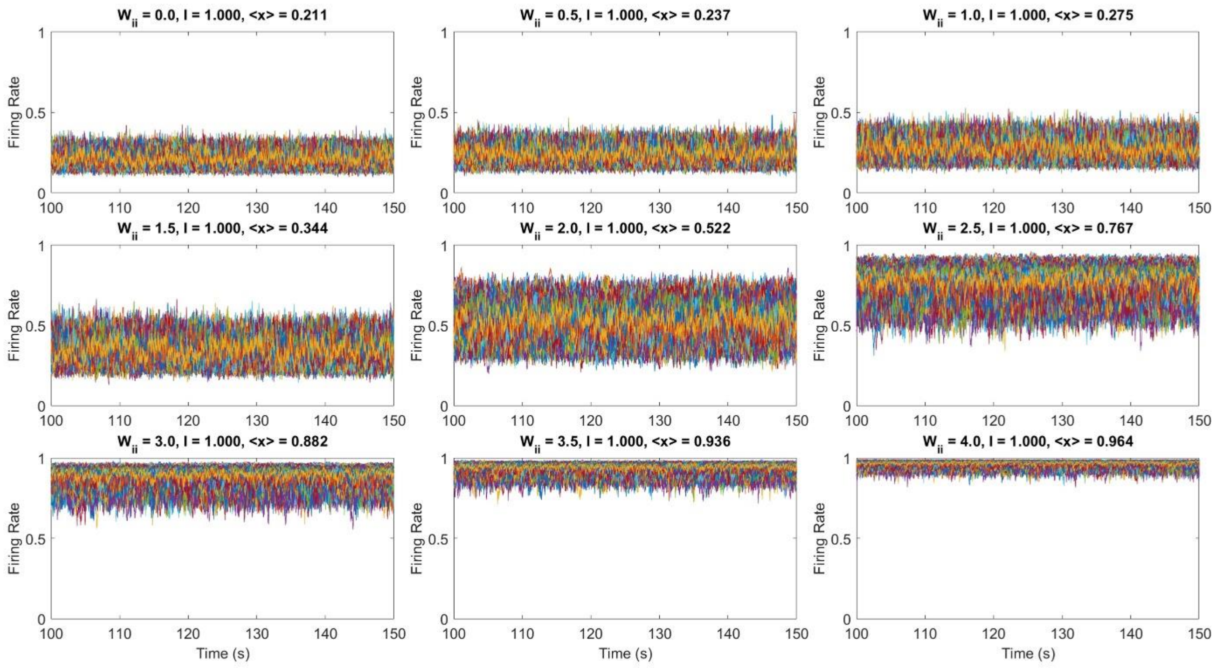


*Supplementary Figure 37: Firing rate time series in one simulation for all regions in stimulated state. $W_{ii}$ denote recurrent connections. $I$ stands for external input (which is set to 3 for the stimulated state) and $< x >$ is the average firing rate across time and regions of interest. Note the saturation of firing rates for $W_{ii} \geq 3$. Each color denotes one region.*

To counter this criticism, we used a set another dynamic equation for $I$ so that for all the values for recurrent connections (see methods), the average resting firing rate is around 0.1 and the average firing rate for stimulated state is around 0.6. This was done for $W_{ii}$ values from 0 to 3. Supplementary figures 38 and 39 show the determined values of $I$, resulting average firing rates and time series of firing rate for one simulation in rest and stimulated states respectively.

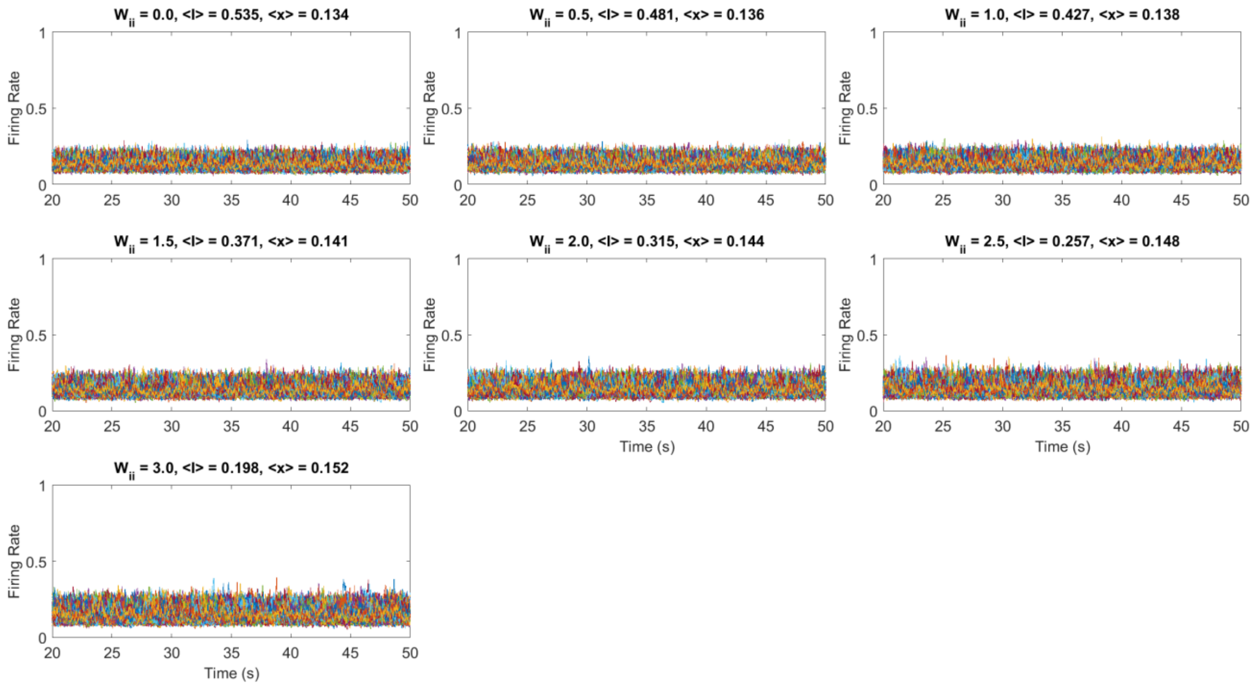


*Supplementary Figure 38: Firing rate time series in one simulation for all regions in resting state with optimized value of external stimulation so that average firing rate will be around 0.1. $W_{ii}$ denote recurrent connections. $I$ stands for external input and $< x >$ is the average firing rate across time and regions of interest. Each color denotes one region.*

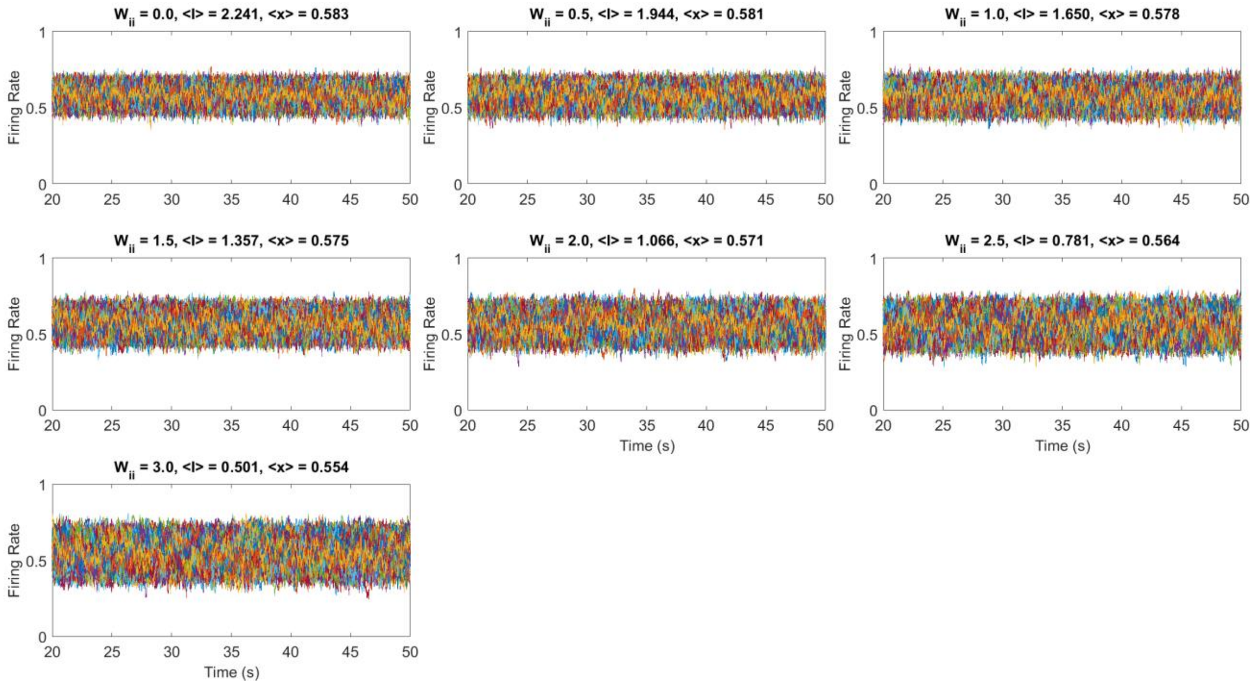


*Supplementary Figure 39: Firing rate time series in one simulation for all regions in stimulated state with optimized value of external stimulation so that average firing rate will be around 0.6. $W_{ii}$ denote recurrent connections. $I$ stands for external input and $< x >$ is the average firing rate across time and regions of interest. Each color denotes one region.*

We reran the simulations with these values of external stimulations to get the rest – stimulated state comparison of $\tau$ values, obtained using the same way in the main paper. Supplementary figure 40 shows the values in all simulations. Note the steady increase of the $\tau$ difference with increasing recurrent connections.

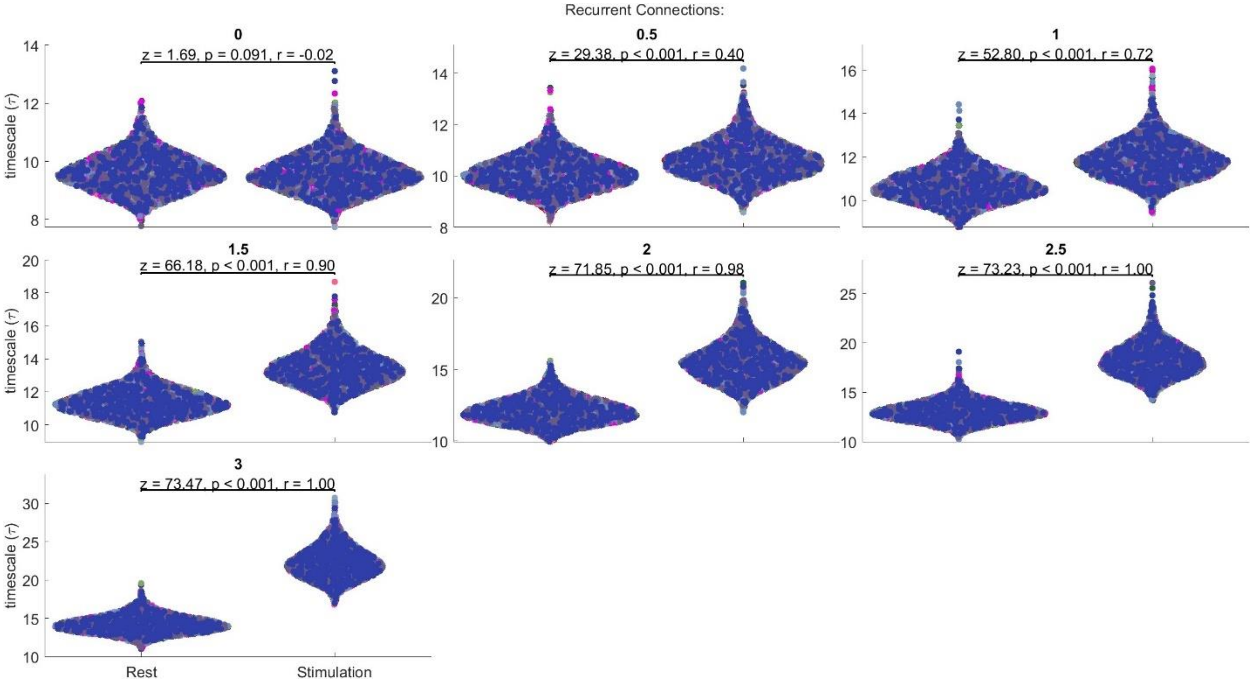


*Supplementary Figure 40: After determining the strength of external stimulation for each value of recurrent connection, we simulate the models again and compare the timescale values in rest and stimulated states. Each dot denotes one region and colors denote simulations.*

## Model with Inhibitory Connections

In order to replicate our results in a model that also includes inhibitory connections, we modified the equations by incorporating one excitatory and one inhibitory population for each region:

$$\tau_E \frac{dx_{E,i}(t)}{dt} = -x_{E,i}(t) + f\left(\sum_{i\neq j} W_{ij} x_{E,i}(t) + C_{EE} x_{E,i}(t) - C_{EI} x_{I,i}(t) + b + I_i(t)\right)$$

$$\tau_I \frac{dx_{I,i}(t)}{dt} = -x_{I,i}(t) + f\big(C_{IE} x_{E,i}(t) - C_{II} x_{I,i}(t) + b\big)$$

Where $x_{E,i}$ and $x_{I,i}$ represent the firing rates of excitatory and inhibitory populations in region $i$ respectively. $C_{ij}$ is the weight matrix of intraareal connections. For numerical simulations, we set $\tau_E$=0.1, $\tau_I$=0.05, $C_{II}$=0.1, $C_{IE}$=0.2, $C_{EI}$=0.8. Remaining parameters are the same as the excitatory-only simulations. By defining $C_{EE}$ as a separate term, we set the diagonal elements of $W$ to 0. $C_{EE}$ corresponds to recurrent excitatory connections and we explore their effect on the dynamics via changing $C_{EE}$ systematically from 0 to 4 in steps of 0.5. Below, we replicate figures 8 and 9 from the main manuscript in this formalism and investigate the ACW changes of excitatory populations.

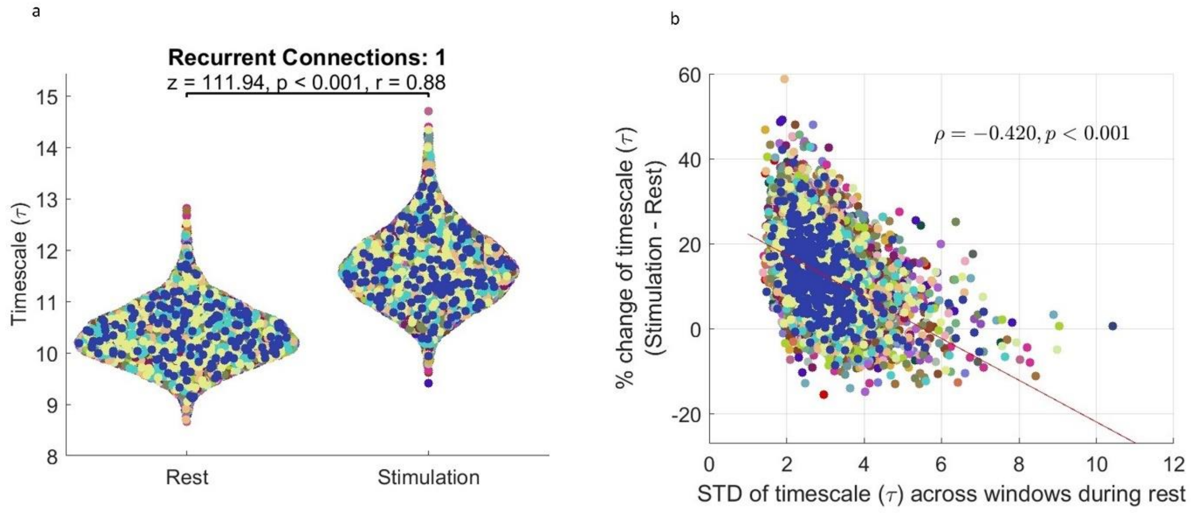


*Supplementary Figure 41. a. Rest – Stimulation change of timescale for the resting model and stimulated model. b. Correlation between rest variability of timescale and rest - task change of timescale.*

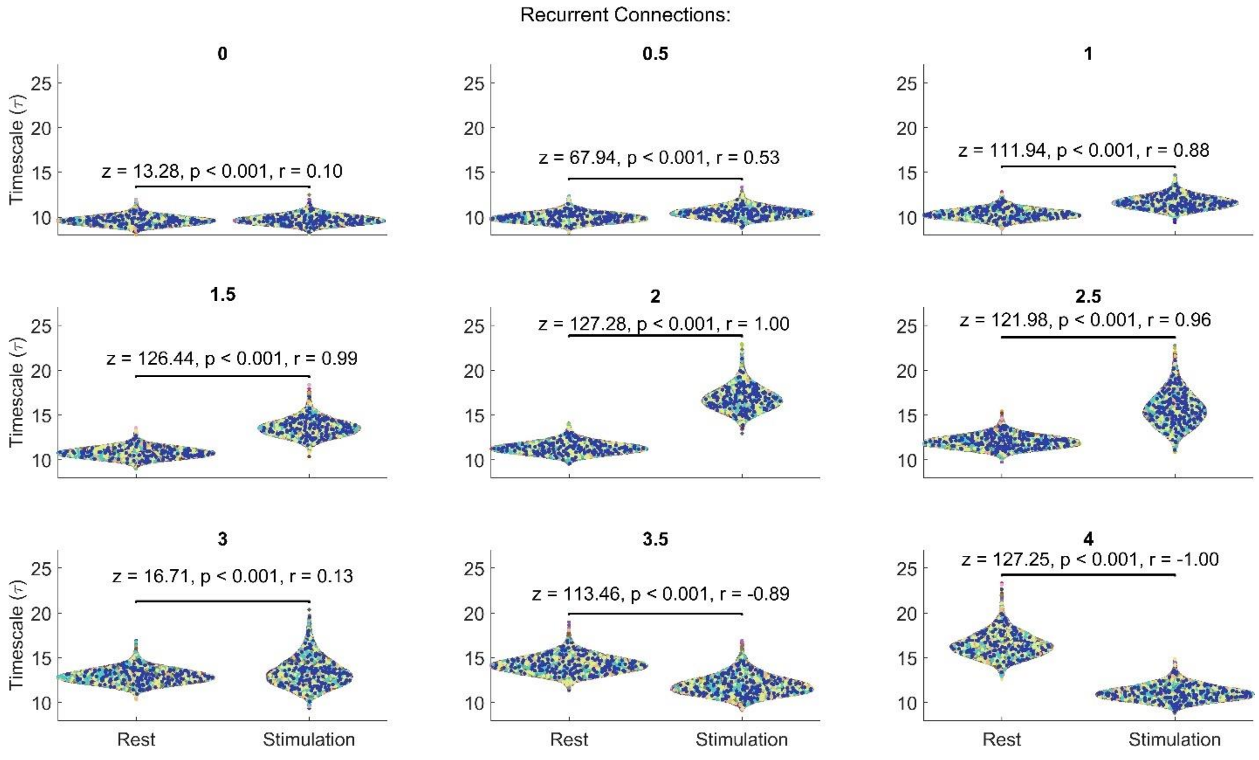


*Supplementary Figure 42. Change of timescale for various values of recurrent connections.*

## *Lyapunov Spectra of the Model*

In order to further characterize the model and assess potential chaoticity of the dynamics, we calculated the Lyapunov spectra for each value of the recurrent connection in both rest and stimulated states. Supplementary figure 43 shows the Lyapunov Spectra.

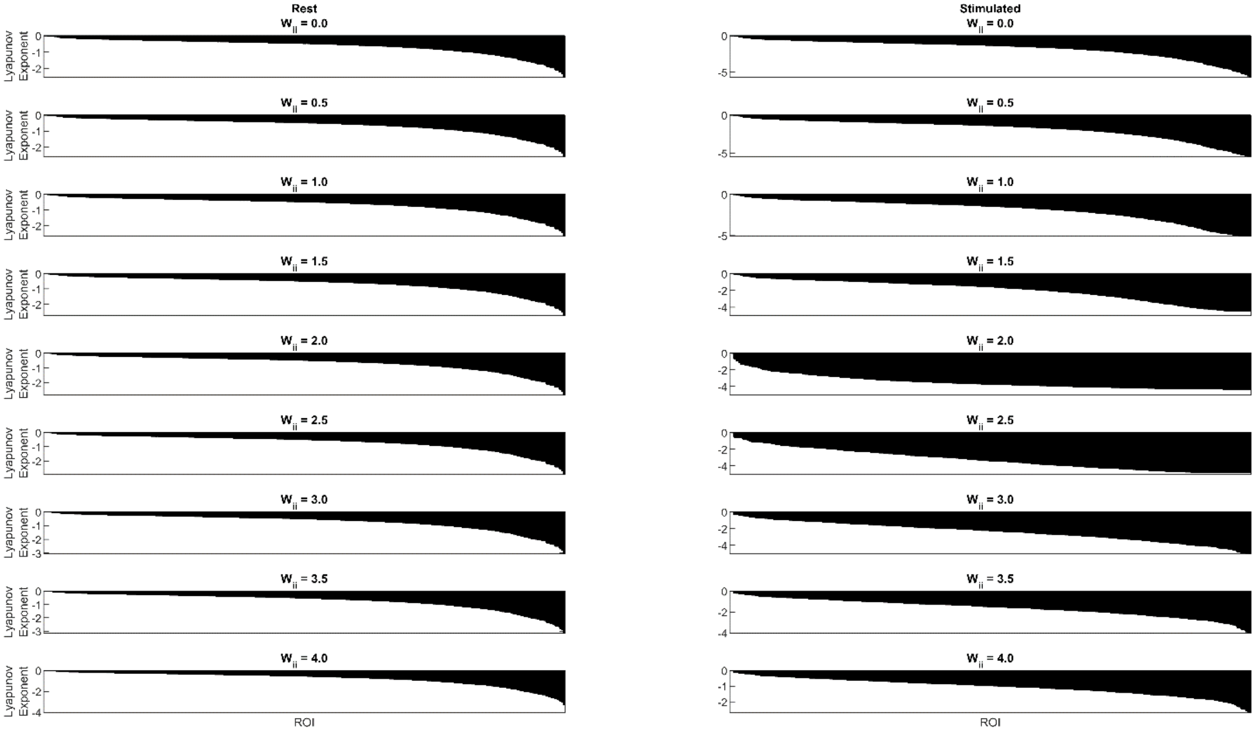


*Supplementary Figure 43. Lyapunov spectra of the model in rest and stimulated states for each value of recurrent connections*

## The effect of network topology on rest – stimulated state change of INTs

By shuffling the edges in the connectivity network, we tested the effect of topological structure on the rest – stimulated state change of INT. We randomly shuffled 20 to 100 percent of edges while keeping the recurrent connections intact 30 times and calculated the $\tau$ values in 10 second sliding windows with no overlap. Supplementary figure 44 shows that in every degree of shuffling, the $\tau$ change remains intact.

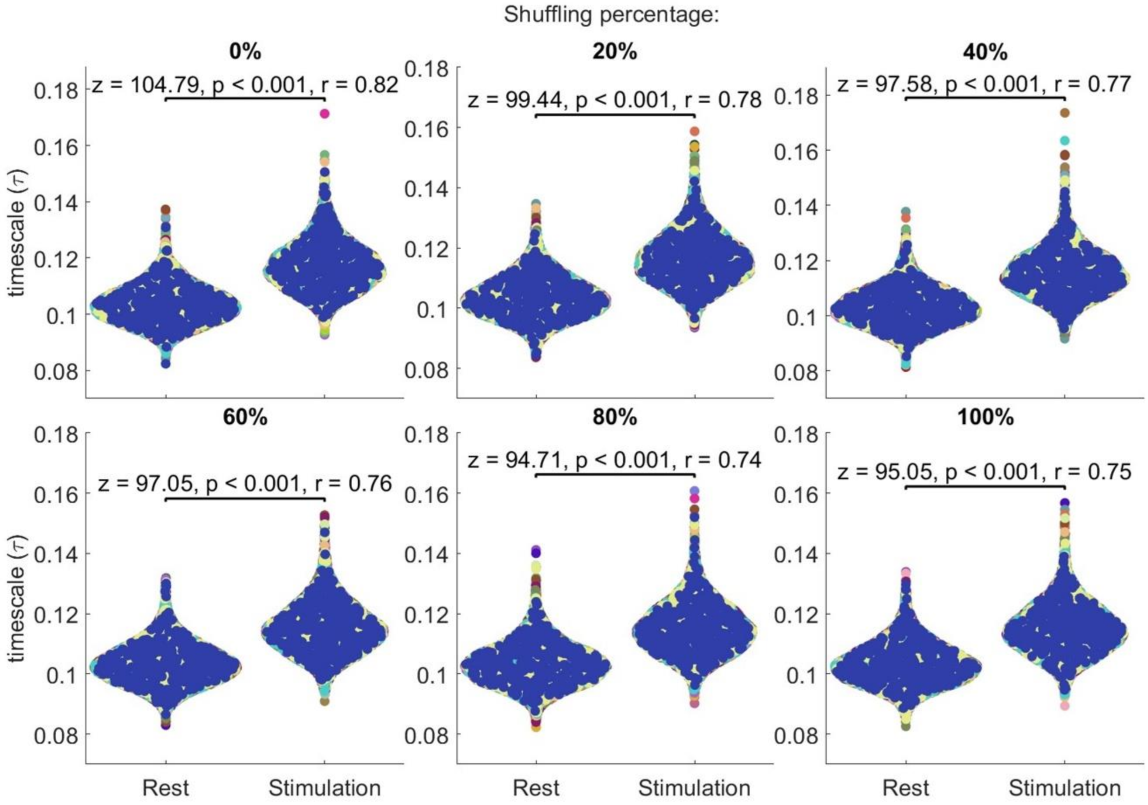


*Supplementary Figure 44: The effect of network topology on change of $\tau$ values. We randomly shuffled the nonzero edges of the connectivity matrix in percentages spanning from 0 to 100 in steps of 20. We compared the $\tau$ values in rest and stimulation states. Each dot denotes one region and colors denote simulations.*
