# Supplementary material for: Flexibility of intrinsic neural timescales during distinct behavioral states
Source: Commun Biol. 2024 Dec 19;7:1667. doi: 10.1038/s42003-024-07349-1 (PMC11659614; doi:10.1038/s42003-024-07349-1)
Supplement: Supplementary file 2 — Reporting Summary [file 42003_2024_7349_MOESM2_ESM.pdf]

nature portfolio

Corresponding author(s): Yasir Catal

Last updated by author(s): Nov 26, 2024

Reporting Summary

Nature Portfolio wishes to improve the reproducibility of the work that we publish. This form provides structure for consistency and transparency in reporting. For further information on Nature Portfolio policies, see our Editorial Policies and the Editorial Policy Checklist.

Statistics

For all statistical analyses, confirm that the following items are present in the figure legend, table legend, main text, or Methods section.

n/a	Confirmed	
☐	☒	The exact sample size (*n*) for each experimental group/condition, given as a discrete number and unit of measurement
☒	☐	A statement on whether measurements were taken from distinct samples or whether the same sample was measured repeatedly
☐	☒	The statistical test(s) used AND whether they are one- or two-sided<br>*Only common tests should be described solely by name; describe more complex techniques in the Methods section.*
☒	☐	A description of all covariates tested
☐	☒	A description of any assumptions or corrections, such as tests of normality and adjustment for multiple comparisons
☒	☐	A full description of the statistical parameters including central tendency (e.g. means) or other basic estimates (e.g. regression coefficient) AND variation (e.g. standard deviation) or associated estimates of uncertainty (e.g. confidence intervals)
☐	☒	For null hypothesis testing, the test statistic (e.g. *F*, *t*, *r*) with confidence intervals, effect sizes, degrees of freedom and *P* value noted<br>*Give P values as exact values whenever suitable.*
☒	☐	For Bayesian analysis, information on the choice of priors and Markov chain Monte Carlo settings
☒	☐	For hierarchical and complex designs, identification of the appropriate level for tests and full reporting of outcomes
☐	☒	Estimates of effect sizes (e.g. Cohen's *d*, Pearson's *r*), indicating how they were calculated

Our web collection on statistics for biologists contains articles on many of the points above.

Software and code

Policy information about availability of computer code

Data collection	No software was used for data collection.
Data analysis	The code used for data analysis will be made available at https://github.com/duodenum96/acw_behavior.

For manuscripts utilizing custom algorithms or software that are central to the research but not yet described in published literature, software must be made available to editors and reviewers. We strongly encourage code deposition in a community repository (e.g. GitHub). See the Nature Portfolio guidelines for submitting code & software for further information.

Data

Policy information about availability of data

All manuscripts must include a data availability statement. This statement should provide the following information, where applicable:

- Accession codes, unique identifiers, or web links for publicly available datasets
- A description of any restrictions on data availability
- For clinical datasets or third party data, please ensure that the statement adheres to our policy

The preprocessed Calcium imaging data can be accessed at https://zenodo.org/records/7968402. The human EEG data are not publicly available due to ethical requirements and privacy concerns.

Research involving human participants, their data, or biological material

Policy information about studies with human participants or human data. See also policy information about sex, gender (identity/presentation), and sexual orientation and race, ethnicity and racism.

Reporting on sex and gender	Out of 27 participants, 10 of them were female assigned at birth. No sex or gender based analysis were performed since these questions were out of the scope of our analysis.
Reporting on race, ethnicity, or other socially relevant groupings	We did not investigate any social, race or ethnicity related questions since these were out of scope of our analysis.
Population characteristics	All participants were right-handed local residents of Ottawa with mean age = 30.3 years.
Recruitment	See original study: Smith D, Wolff A, Wolman A, Ignaszewski J, Northoff G. Temporal continuity of self: Long autocorrelation windows mediate self-specificity. Neuroimage. 2022 Aug 15;257:119305. doi: 10.1016/j.neuroimage.2022.119305. Epub 2022 May 11. PMID: 35568347.
Ethics oversight	The experiments were approved by the local ethics board (REB NUMBER: 2016004) of the University of Ottawa Institute of Mental Health Research.

Note that full information on the approval of the study protocol must also be provided in the manuscript.

Field-specific reporting

Please select the one below that is the best fit for your research. If you are not sure, read the appropriate sections before making your selection.

☒ Life sciences ☐ Behavioural & social sciences ☐ Ecological, evolutionary & environmental sciences

For a reference copy of the document with all sections, see nature.com/documents/nr-reporting-summary-flat.pdf

Life sciences study design

All studies must disclose on these points even when the disclosure is negative.

Sample size	We used data from previous studies. For Calcium Imaging, sample size is 5 mice. Human EEG dataset consists of 27 healthy participants.
Data exclusions	four participants were excluded from EEG analysis due to the noisy channels, one participant was excluded due to the absence of alpha peak and finally one more subject was excluded due to technical issues relating to data acquisition.
Replication	We did not take any measures to replicate the findings we report.
Randomization	Not applicable
Blinding	Not applicable

Reporting for specific materials, systems and methods

We require information from authors about some types of materials, experimental systems and methods used in many studies. Here, indicate whether each material, system or method listed is relevant to your study. If you are not sure if a list item applies to your research, read the appropriate section before selecting a response.

Materials & experimental systems

n/a	Involved in the study	
☒	☐	Antibodies
☒	☐	Eukaryotic cell lines
☒	☐	Palaeontology and archaeology
☐	☒	Animals and other organisms
☒	☐	Clinical data
☒	☐	Dual use research of concern
☒	☐	Plants

Methods

n/a	Involved in the study	
☒	☐	ChIP-seq
☒	☐	Flow cytometry
☒	☐	MRI-based neuroimaging

Animals and other research organisms

Policy information about studies involving animals; ARRIVE guidelines recommended for reporting animal research, and Sex and Gender in Research

Laboratory animals	We used data previously collected for the paper Shahsavarani, S. et al. Cortex-wide neural dynamics predict behavioral states and provide a neural basis for resting-state dynamic functional connectivity. Cell Reports 42, (2023). Paraphrasing from that paper:<br><br>Transgenic male adult Thy1-jRGECO1a mice expressing red-shifted calcium indicators were used in this study. The mice were bred from the line Tg(Thy1-jRGECO1a)GP8.20Dkim/J, purchased from the Jackson Laboratory. The mice were three months old when the experiments began.
Wild animals	The study did not involve any wild animals.
Reporting on sex	All mice were male. For additional details, see the original paper hahsavarani, S. et al. Cortex-wide neural dynamics predict behavioral states and provide a neural basis for resting-state dynamic functional connectivity. Cell Reports 42, (2023).
Field-collected samples	No field collected samples were used.
Ethics oversight	We used the publicly available data https://zenodo.org/records/7968402. Our analyses conforrm to the local ethics committee of University of Ottawa Institute of Mental Health Research with REB: 2021002.

Note that full information on the approval of the study protocol must also be provided in the manuscript.

Plants

Seed stocks	*Report on the source of all seed stocks or other plant material used. If applicable, state the seed stock centre and catalogue number. If plant specimens were collected from the field, describe the collection location, date and sampling procedures.*
Novel plant genotypes	*Describe the methods by which all novel plant genotypes were produced. This includes those generated by transgenic approaches, gene editing, chemical/radiation-based mutagenesis and hybridization. For transgenic lines, describe the transformation method, the number of independent lines analyzed and the generation upon which experiments were performed. For gene-edited lines, describe the editor used, the endogenous sequence targeted for editing, the targeting guide RNA sequence (if applicable) and how the editor was applied.*
Authentication	*Describe any authentication procedures for each seed stock used or novel genotype generated. Describe any experiments used to assess the effect of a mutation and, where applicable, how potential secondary effects (e.g. second site T-DNA insertions, mosiacism, off-target gene editing) were examined.*
